# Relative importance of climate versus socio-environmental development changes to 2050 in rural coastal Bangladesh—a system analysis

**DOI:** 10.1007/s10113-025-02511-9

**Published:** 2026-02-07

**Authors:** Attila N. Lázár, Robert J. Nicholls, Craig W. Hutton, Andres Payo, Helen Adams, Anisul Haque, Derek Clarke, Mashfiqus Salehin, Alistair Hunt, Andrew Allan, W. Neil Adger, M. Munsur Rahman, Roland Smith

**Affiliations:** 1 School of Geography and Environmental Science, University of Southampton, Southampton, SO17 1BJ UK; 2 Tyndall Centre for Climate Research, University of East Anglia, Norwich, NR4 7TJ UK; 3 School of Engineering, Faculty of Physical Sciences and Engineering, University of Southampton, Southampton, SO16 7QF UK; 4 British Geological Survey, Keyworth, Nottingham, NG12 5GG UK; 5 Department of Geography, King’s College London, London, WC2B 4BG UK; 6 Bangladesh University of Engineering and Technology, Dhaka, 1000 Bangladesh; 7 Economics, University of Bath, Bath, BA2 7AY UK; 8 UNESCO Centre for Water Law, Policy and Science, University of Dundee, Dundee, DD1 4HN UK; 9 Faculty of Environment, Science and Economy, University of Exeter, Exeter, EX4 4RJ UK

**Keywords:** Integrated assessment model, Climate change, Policy options, Human wellbeing, Coastal adaptation

## Abstract

Climate change and development are having a profound influence on the integrity of coastal Bangladesh’s people, economy and ecology with climate change widely considered the dominant driver. Our bespoke integrated assessment model (IAM) simulates these changes for rural areas by coupling physical models with economic models, considering shocks like cyclone landfalls, and utilising substantial primary/secondary datasets and stakeholder-derived future scenarios. As such, this study assesses the Bangladesh coastal system’s sensitivity to climate change and policy decisions to 2050. The IAM results suggest that flooding and salinisation have high sensitivity to climatic and environmental drivers, and agricultural productivity is strongly dependent on farming methods and investment in better crop varieties to counteract these changes. Inequality, poverty and GDP per capita are more sensitive to non-climatic drivers. Nonetheless, extreme climatic conditions do impact livelihoods due to economic damage with variable effects across different household types. At least over the next 25 years, development choices (i.e. concrete adaptation actions) will have a similar if not stronger influence on livelihoods and economic wellbeing in rural coastal Bangladesh than climate-driven environmental change. This IAM approach supports decision makers to identify sustainable development pathways that address both current socio-environmental vulnerability as well as develop a more climate-resilient future.

**Supplementary Information:**

The online version contains supplementary material available at 10.1007/s10113-025-02511-9.

## Introduction

Coastal delta areas comprise low-lying, complex systems which occupy less than 1% of global land surface area but are home to up to 500 million people (Anthony et al. 2024). Climate is regularly portrayed as the dominant driver of change in delta regions, particularly sea-level rise (SLR) (Nienhuis et al. 2023), but contemporary deltas are complex systems, shaped by multiple interacting socio-environmental and biophysical drivers (Nicholls et al. 2020; Eslami et al. 2025). The scale of the challenge for delta management is immense with an urgent need for the establishment of robust governance frameworks to meet international commitments such as the Sustainable Development Goals and the adaptation goal in the Paris Agreement on climate. As a result, more holistic Adaptive Delta Management processes are being developed to promote adaptation and development (Seijger et al. 2017; Zevenbergen et al. 2018). However, such approaches are hindered by the complex interactions in delta systems which make it difficult to understand how multiple drivers and responses interact and their relative significance on development trajectories (Schmitt and Minderhoud 2023; Eslami et al. 2025). The inherent complexity of such planning (Korbee et al. 2019) is a barrier to evidence-based prioritisation and implementation of development choices, including adaptation. This study addresses this challenge by utilising an Integrated Assessment Model (IAM) to elucidate and analyse relationships between key factors which shape the socio-environmental status of delta systems.

The Bangladesh coast in the Ganges-Brahmaputra delta, for example, is highly exposed to hazards associated with climatic shocks and environmental degradation, including incidence of tropical cyclone, storm surge, tidal inundation, waterlogging, salinity intrusion and erosion (GED 2018; Uddin et al. 2019; Barbour et al. 2022; Sultana and Luetz 2022; Kabir et al. 2025). The inter-related processes of monsoon precipitation, storm surge and tidal inundation render the coastal zone prone to flood events, which have the potential to cause severe damage to agriculture, aquaculture and associated coastal livelihoods (Haque and Nicholls 2018). Frequent destructive cyclones (e.g. Sidr in 2007, Aila in 2009, Amphan in 2020) also result in substantial loss of life and major economic damage (Elahi et al. 2025; Nazrul et al. 2025; van Schie et al. 2025). Coastal Bangladesh is also highly exposed to SLR (Huq et al. 1995; World Bank 2010; Nicholls et al. 2018; Elahi et al. 2025), and combined with projections of an increase in the frequency of more intense cyclones (Nazrul et al. 2025), this is likely to result in an increase in storm surge, coastal flooding and economic losses (Haque and Nicholls 2018; Duijndam et al. 2022; Smith et al. 2023; Becker et al. 2024).

Impacts associated with climate change have the potential to severely affect the lives and livelihoods of Bangladesh’s coastal population (Aryal et al. 2020; BBS 2022; Das et al. 2021; Hasan and Kumar 2020). As such, the Government of Bangladesh, alongside international agencies and NGOs, has implemented a number of policies and schemes which address the coastal zone’s vulnerability to extreme events, including the expansion of a cyclone shelter network alongside the development of the community-focused Cyclone Preparedness Programme (CPP) and government-level Comprehensive Disaster Management Programme (CDMP) (Haque et al. 2022; MoEFCC 2022; Alam 2023). Collectively, these efforts have reduced flood-related mortality in high-risk areas by about 70% (Majlingova and Kádár 2025). Latterly, the Bangladesh Delta Plan 2100 was formulated to address population growth, food insecurity, recurrent hazards and groundwater stress while promoting development and prosperity. Founded on the principles of Adaptive Delta Management, it promotes an integrated, long-term approach to water management and governance, linking immediate priorities with future challenges, and is aligned with national development goals (GED 2018; Kulsum et al. 2021). Within these overarching policy frameworks, the Department of Agricultural Extension is working with farmers to introduce climate-smart agriculture practices (Hasan et al. 2018; Islam et al. 2021). Hence, climatic change is only part of a wider range of socio-environmental drivers in the Ganges-Brahmaputra delta system (Dasgupta et al. 2013; Fernandes et al. 2016; Roy et al. 2017; Nicholls et al. 2018).

Several studies have undertaken simple risk and vulnerability assessments focused on coastal Bangladesh, using climate scenarios to assess plausible future vulnerability states (Alamgir et al. 2018; Mehvar et al. 2019; Uddin et al. 2019; Murshed et al. 2022). They indicate that vulnerability is dependent on policy decisions and natural resource management as well as hazards. Importantly, these types of assessments often take a narrow, sectoral view which may miss important outcomes due to interactions with wider changes. With the development aspirations of the Bangladesh Government exemplified by the Bangladesh Delta Plan 2100 and other initiatives, it is important to understand these interactions. What limitations are imposed on development by climate change and equally what opportunities can be plausibly unlocked and realised by development and policy actions? In particular, what are the implications for the biophysical environment and the residents of coastal Bangladesh? These unknowns comprise a significant and important gap in our understanding. Addressing this demands the development of new methods and approaches to assess holistically the dynamic and complex interactions that characterise delta systems.

Such analysis requires a system rather than a sectoral understanding. System dynamics models (SDMs) comprise one approach—simple representations of complex systems encompassing multiple sectors to understand and analyse changes over time and simulate ‘what-if’ scenarios (Borgomeo et al. 2017; Hossain et al. 2020). However, when more detailed processes and spatial complexity are needed, as in representing complex human-environment coupling in coastal Bangladesh, IAMs are better suited. IAMs provide a systems approach to consider the interaction of multiple change factors without necessarily prioritising one over the other. They go beyond simple risk assessment by considering substantial process-details and have generally been designed to realistically analyse real-world system problems including people and the natural world and thus support evidence-based action (Jakeman and Letcher 2003; Kelly et al. 2013). Relevant examples include acid rain (Hordijk and Kroeze 1997), air quality (Schöpp et al. 1998), climate change risks (Harrison et al. 2019), national infrastructure systems development (Hall et al. 2016), climate-induced immobility (Benveniste et al. 2022), agriculture and climate change (Jafino et al. 2021; Huang et al. 2022) and development/adaptation choices (van Vuuren et al. 2015; Lázár et al. 2020b). As such, we employ an IAM to analyse the future development of coastal Bangladesh.

The aim of this paper is to analyse the gap in our understanding of the relative importance of different drivers of the future socio-environmental status of coastal Bangladesh, and to compare the magnitude of the impacts of climatic (exogenous) drivers and the impacts of human/development (endogenous) drivers. We utilise an existing and already validated IAM to analyse the sensitivity of the rural Bangladesh delta system to policy decisions and climatic scenarios across a range of plausible scenarios to 2050. Rural areas and their ecosystem services are expected to be especially sensitive to climate and environmental changes. The specific objectives are as follows: (i) to quantify the future trajectories of key environmental and socio-economic indicators, (ii) to examine the sensitivity of key environmental and socio-economic indicators to plausible climatic and socio-environmental drivers and (iii) to identify the influences of climatic and socio-economic changes on different population groups and poverty. We stress that the sensitivity results presented here are not designed to be used as projections.

## Methods

### Study area

**Fig. 1:**
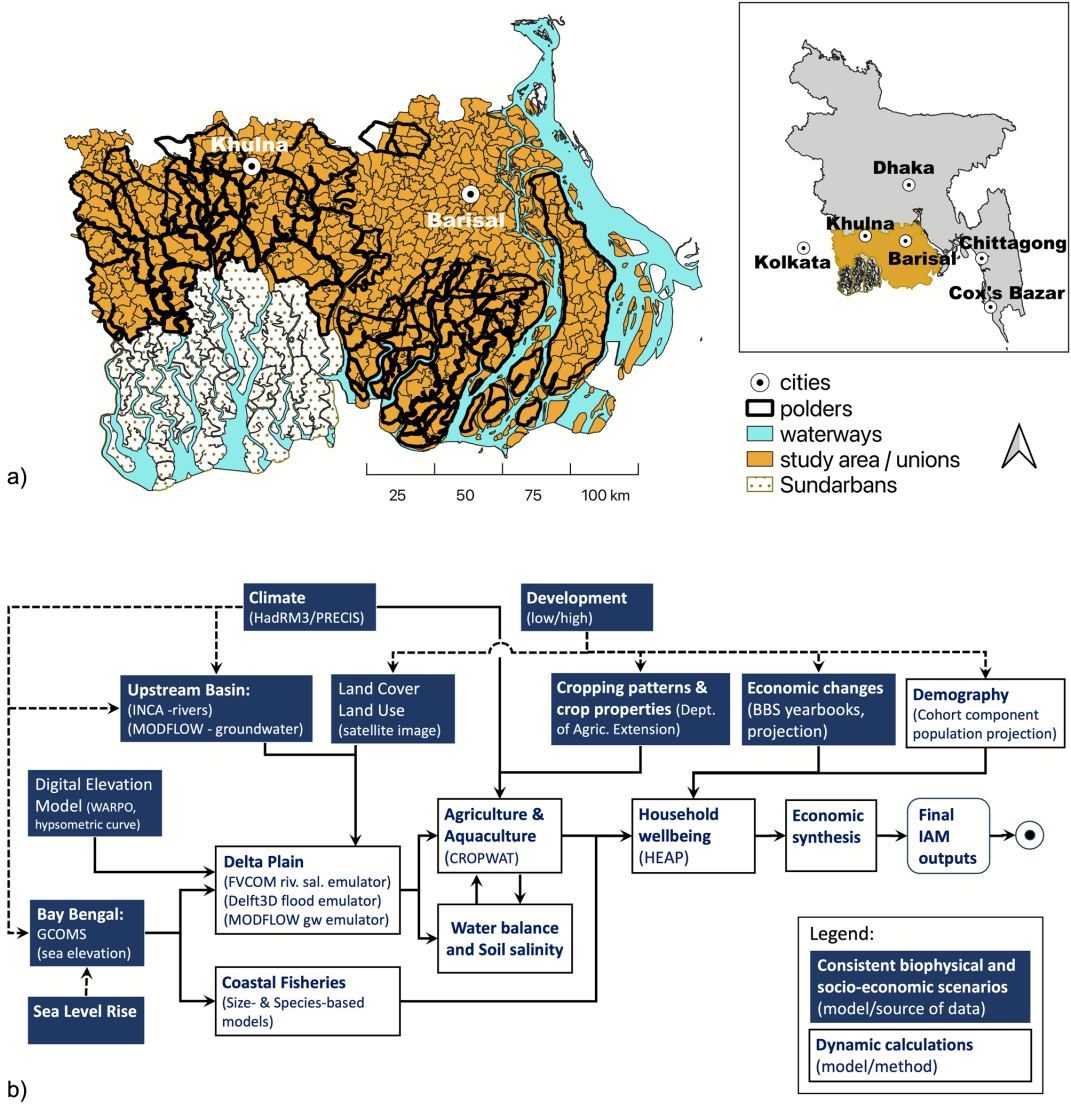
Map of the study area (**a**) and overview of the Delta Dynamic Integrated Emulator Model showing consistent biophysical and socio-economic input scenarios and dynamic calculations. Input sources and calculation methods are in brackets (**b**)

This study focuses on coastal Bangladesh where large-scale engineering interventions are widespread (Fig. 1a). There are 105 polders with ~ 5000 km in our study area influencing, amongst others, flooding, salinisation and sedimentation dynamics (Barbour et al. 2022). (A polder is a low-lying area of land that was once marshy/waterlogged but has been reclaimed by building embankments around it and providing drainage. Polders are generally used to facilitate agriculture, as in Bangladesh.)

Around 38 million people live in the nationally defined coastal Bangladesh, an area of 47,201 km^2^ that contributes 25% to the country’s economy. This includes agriculture and fishing (16% of delta GDP), service industries (22% of delta GDP), trade and transport sector (31% of delta GDP) and industries and construction (30% of delta GDP) (Nicholls et al. 2020). Here we analyse coastal South-West and South-Central Bangladesh southwest of the Ganges-Padma, with 14 million inhabitants on 18,850 km^2^ in a largely rural setting (Fig. 1a).

In our study area, about 70% of rural households have mixed income sources (Figure S3) and about 85% of households engage in agriculture (Adams et al. 2016a). Some 56% of households are functionally landless, but their income relies on wage labour in the agricultural sector (BBS 2012). In addition, fishing, shrimp aquaculture, salt production and tourism are significant economic activities (Abedin et al. 2012; Rahman et al. 2020). Mangrove forest comprises around 10% of the study area (mainly in the Sundarbans), offering important natural resources to local communities.

### The delta dynamic integrated emulator model (ΔDIEM)

We applied an IAM to rural areas of coastal Bangladesh—The Delta Dynamic Integrated Emulator Model (ΔDIEM) (Fig. 1b). ΔDIEM is a tightly coupled integrated assessment platform capturing a range of biophysical (climate, upstream hydrology, sea elevation, fisheries) and socio-economic (demography, economy, land use, household coping behaviours) drivers and factors, including delta planning dimensions (Nicholls et al. 2016, 2018; Lázár et al. 2019). The aim of the integration was to (i) capture the most important physical and social aspects of coastal Bangladesh in a harmonised scenario framework, (ii) utilise detailed model- or survey-based background studies of these aspects and (iii) integrate these detailed studies on the same platform using various complexity representations (look-up tables, emulators, process-based models) for rapid simulation at the highest spatial/temporal resolution possible. Therefore, ΔDIEM encapsulates these drivers to assess the cumulative effect of changes on the well-being and poverty of the coastal population (Fig. 1b) (Lázár et al. 2020a). ΔDIEM outputs include flooded area, soil salinisation, agriculture productivity, livelihood potential and human well-being, as well as income inequality, poverty and GDP, disaggregated by sub-populations. ΔDIEM was developed via sustained engagement with diverse Bangladeshi stakeholders at multiple levels of influence, ranging from national government and development agencies to local NGOs and academic experts (Nicholls et al. 2018; Allan et al. 2022). A large number of structural and policy interventions (e.g. embankment management, new crops, subsidies, loan structures, etc.) can be tested within a robust scenario framework, as well as quantify different development trajectories including trade-offs (Hutton et al. 2018; Rahman et al. 2019). Thus, ΔDIEM offers several advantages compared to earlier assessments by including the following: (i) climate, environmental and socio-economic drivers, (ii) interactions between model components including scale harmonisation, (iii) coastal management and governance dimensions and (iv) household coping and adaptation at the local level for multiple archetypal household types. Submodules of ΔDIEM are individually designed and tested against observations first, then integrated and refined with the other modules. Detailed information on inputs and equations is in the Supplementary Information, but here we provide a comprehensive overview.

Drivers and processes outside the coastal zone are boundary conditions of the model, expressed as user-specified input scenarios. These include transient climate scenarios (approximately RCP6) (Caesar et al. 2015), simulation study outputs for upstream hydrology using the INCA model (Whitehead et al. 2015), sea levels based on Church and Clark (2013) and the GCOMS model, fisheries using a model system (Fernandes et al. 2016), demography calculations using the cohort component method (Szabo et al. 2016) and economic trend assumptions based on Bangladesh Bureau of Statistics yearbooks (Hunt 2018) for the period of 1981–2050. Our land use and land cover inputs are based on the remote sensing analysis of historical trends (Mukhopadhyay et al. 2018) and stakeholder narratives about the future (Allan et al. 2022). These inputs are look-up tables from which the appropriate time series is selected for each model run.

The biophysical aspects of the coastal zone are captured dynamically with both statistical emulators and process-based calculations. Coastal hydrology is based on a library of model runs of detailed three-dimensional, physics-based models such as Delft-3D (surface water quantity), FVCOM (river salinity) and MODFLOW-SEAWAT (groundwater quantity and quality). Such models are physics-based and capture the coastal processes realistically but are computationally unrealistic to run in an IAM. Hence, ΔDIEM uses statistical emulators (Payo et al. 2017) to simplify these complex models. The emulators combine the Partial Least Square regression with Canonical Correlation Analysis to represent the simulated spatial and temporal dynamics of these complex models by statistically establishing a relationship between inputs (e.g. incoming river discharge, daily sea elevation statistics) and outputs (e.g. river elevation, inundated area, mean inundation depth). Emulators are useful when the focus of the analysis is not on the actual mechanics of changes but rather the changes in the outputs.

ΔDIEM represents two important biophysical issues that strongly shape rural livelihoods and the economy in coastal Bangladesh: soil salinisation and farming. The soil salinisation process is complex with drivers of precipitation, evapotranspiration, capillary rise, infiltration, deep percolation, surface water drainage, flooding and irrigation. ΔDIEM fully couples these drivers in a water and salt-balance calculation (Payo et al. 2017). The soil water content is, for example, governed by porosity, depth of the root zone (plant growth cycle-driven), and the water inputs and discharges, and ΔDIEM continuously account for these spatial and temporal changes. The farming module is based on the FAO’s CROPWAT model simulating crop development based on available water (Allen et al. 1998), and here it is extended with salt-, temperature- and flood-water-related limitations and atmospheric fertigation to simulate both traditional agriculture and pond-based aquaculture (Lázár et al. 2015). The soil and crop processes are represented with detailed process-based equations and fully coupled with inputs and other modules in ΔDIEM (Payo et al. 2017).

ΔDIEM links biophysical changes to livelihood potential and simulates household well-being with an agent-based approach called HEAP (Lázár et al. 2020a). This model was built on Household Income and Expenditure Survey data and a novel household survey (Adams et al. 2016a, b) and simulates the well-being trajectories of 36 household types. The household survey collected longitudinal data from 1586 households on material, subjective and health dimensions of well-being in the context of the use of ecosystem services. Out of these households, 1478 could be interviewed in all seasons; thus, these consistent households are the bases of HEAP. These observed households are grouped in ΔDIEM into types based on observed seasonal variations of six occupation types: (i) farming (agriculture/aquaculture/farm animals), (ii) farm labour, (iii) fishing, (iv) forest goods collection, (v) manufacturing and (vi) small business activities. ΔDIEM simulates farming, farm labour requirements, and fish catches dynamically. Forest goods from mangroves are a static input as these basic services are assumed to be sustained to 2050 (Mukhopadhyay et al. 2015; Payo et al. 2016). Finally, non-natural resource-based livelihoods (businesses, services and manufacturing) are represented as a simple input scenario (Hunt 2018).

The multidimensional well-being of the households is expressed through a set of simulated expenditure levels, which provide poverty indicators over income or assets (see Supplemental Material). They indicate if basic needs are met and if households can survive seasonal poverty using coping mechanisms (Falkingham and Namazie 2002). ΔDIEM matches the income and fixed livelihood costs to the affordable level for household expenditure (e.g. food, education, health, etc.) for each archetypal household (Lázár et al. 2020a). This optimisation includes different coping mechanisms (use cash savings, selling assets, getting loan(s), reduced expenditure, temporary agriculture labouring) and safety nets (postponing loan repayment and support from friends and family). The optimisation algorithm searches for a solution that (1) minimises the number of members engaging in labour work, (2) minimises the number of coping strategies, but (3) maximises the financial capacity level while maintaining a minimum net savings. Poverty indicators (hungry periods, income inequality, GDP/capita) are estimated at household/district/delta scales.

The simulation period is 1985–2050, where the 1985–2004 period is the model spin-up, the 2005–2014 period is the baseline and the analysis focuses on 2015–2050. ΔDIEM produces results for 653 ‘Unions’, which are the smallest administrative unit in Bangladesh. On average, each Union comprises up to 9 villages with ~ 21,000 people over 26 km^2^. The cities of Khulna and Barisal with 1 million inhabitants in total are excluded from our analysis given their urban nature.

Biophysical calculations have a daily/Union scale. Demography is simulated with a 5-year timestep at district scale that is then downscaled to monthly/Union scale. Household calculations are done for each archetype at monthly/Union scale. Protected (i.e. poldered) and non-protected areas are treated separately in ΔDIEM.

Validation of ΔDIEM comprised three boundary adequacy tests: (1) model structure verification, (2) behaviour reproduction tests, and (3) behaviour sensitivity tests. The coastal hydrological and soil salinity emulators are in good agreement with the high fidelity models of Delft-3D, FVCOM and MODFLOW-SEAWAT and field observations (Payo et al. 2017). Crop simulations were compared with observed administrative totals showing good fit both spatially and temporally (Lázár et al. 2015). The household well-being outputs were evaluated against national and regional observations. Errors were not quantified due to the spatial miss-match, but the simulated values qualitatively captured the magnitude and trend of the observations increasing confidence in the overall behaviour of ΔDIEM (Lázár et al. 2020a).

Model limitations and validation are described in detail in the Supplemental Material.

### Design of the sensitivity analysis

To explore a range of combinations of environmental change and different policy choices in the study area, 62 ΔDIEM simulations were conducted (Table S1). The ensemble consisted of 36 climate and socio-economic scenarios (co-developed with stakeholders (Allan et al. 2022)) and a further 24 simulations to explore the sensitivity of individual drivers. The biophysical drivers include cyclones, SLR and changes in climate and freshwater availability (e.g. precipitation, air temperature and river discharge). The socio-economic drivers include population change, economic growth, land cover change, farm management, crop varieties and embankment maintenance. Simulated sequences of cyclones based on historic data were applied as plausible scenarios of climate-induced shocks, with modified (higher and lower) cyclone frequencies in the sensitivity analyses (Table S2).

We analyse two plausible, but contrasting, internally consistent futures focused on trends in poverty and environmental quality over time (Hunt 2018; Allan et al. 2022). The ‘Positive World’ represents improving trends, while the ‘Negative World’ represents deteriorating trends to 2050 (Table 1). In the Positive World, economic development to 2050 is relatively high (2.5% economic growth nationally per annum). In the Negative World, there is lower assumed economic development (0.6% per annum), impacting on flood defence management and farm management opportunities resulting in the gradual outmigration of the population. The two Worlds are similar in climate terms, reflecting projections to 2050 (see IPCC 2021). For SLR, the worlds are distinct with higher SLR assumed in the Negative World, reflecting a high impact, low likelihood future. More details of the key parameters are provided in Table 1 and Table S1.

**Table 1:** Mid-Century scenario descriptions of the Positive and Negative Worlds for coastal Bangladesh (Hunt 2018; Lázár et al. 2018)

	Scenario descriptions (base period: 2000–2015; mid-century: 2040–2055)	
	‘Positive World’	‘Negative World’
Relative sea-level rise (relative to year 2000)	Slow: 38.5 cm, reflecting median scenarios	Fast: 73.5 cm, reflecting a high-end SLR scenario^47^
Temperature rise	+ 1.8 °C (+ 1.5 °C in monsoon period; + 2.2 °C in dry season period)	+ 1.9 °C (+ 1.5 °C in monsoon period; + 2.5 °C in dry season period)
Precipitation changes	Warmer and relatively drier (−22% annual precipitation compared to base period; −19% total monsoon rain and −53% total dry season rainfall)	Warmer and relatively wetter (−4% annual precipitation compared to base period; −2% total monsoon rain and −16% total dry season rainfall)
Cyclones	Current occurrence pattern continues. Strong cyclones (140 + km/h wind speed) and weak cyclones (80–140 km/h wind speed) with return periods of 0.4 and 4.2 per decade, respectively, with random landfalls along the coast	
Population	Constant at 14 million	Decreasing to 11 million, reflecting net outmigration
Embankments	Embankments maintained at design condition (height and strength)	Slow, steady decline in embankment height (−3 cm per year)
Farming and agriculture	New crop types increase unit production (+ 20%). Salinity tolerance of rice increases linearly from 6 to 13 dS/m	Traditional crop types, no growth in unit production. Salinity tolerance of rice stays at 6 dS/m
Economic growth	Higher growth (2.5% per year)	Slower growth (0.6% per year)
Governance	Integrated policies, effective implementation and good maintenance	Poor planning, ineffective implementation and poor maintenance

Further details on methods and input scenarios are found in the Supplementary Document.

To fully analyse system sensitivities, a further 26 scenarios were tested, altering one driver at a time for the Positive and Negative world scenarios (see Table S1 for further details):

- **Climate**: climate change and ‘no climate change’ time series
- **Sea-level rise**: low and high
- **Cyclone frequencies**: historic, + 50% increase, −50% decrease (see Table S2)
- **Embankments**: maintained heights, declining heights
- **Socioeconomic development**: More sustainable, Business as Usual and Less Sustainable defined and varied independently for (i) Population size, (ii) Economic development trends, (iii) Land cover changes and (iv) Farming practices.

The sensitivity of key outputs to the drivers were quantified through the 62 model simulations (Table S1). This analysis is designed to provide insights on the relative importance of a diverse and integrated set of drivers and does not provide projections of the future.

### Selected livelihood indicators

Six indicators were selected to illustrate changes in biophysical, ecological and socio-economic components of the socio-environmental system in coastal Bangladesh to 2050. These are:

- Inundated area (the amount of land flooded each year due to fluvial and coastal floods)
- Soil salinity (mean, due to saltwater inundation and poor irrigation practices)
- Total rice production (impacts of water availability, soil salinity and heat stress)
- Poverty rate (percentage of the population below the national poverty line)
- Income inequality (GINI index)
- GDP/capita (total income divided by the total population).

The trends in each indicator were grouped under the Positive and Negative World trajectories.

## Results

### Future trajectories of key environmental and socio-economic indicators

There is a clear distinction in most of the key indicators between the Positive and Negative Worlds, where the Negative World had worse outcomes (except Soil salinity) as explained below.

**Fig. 2:**
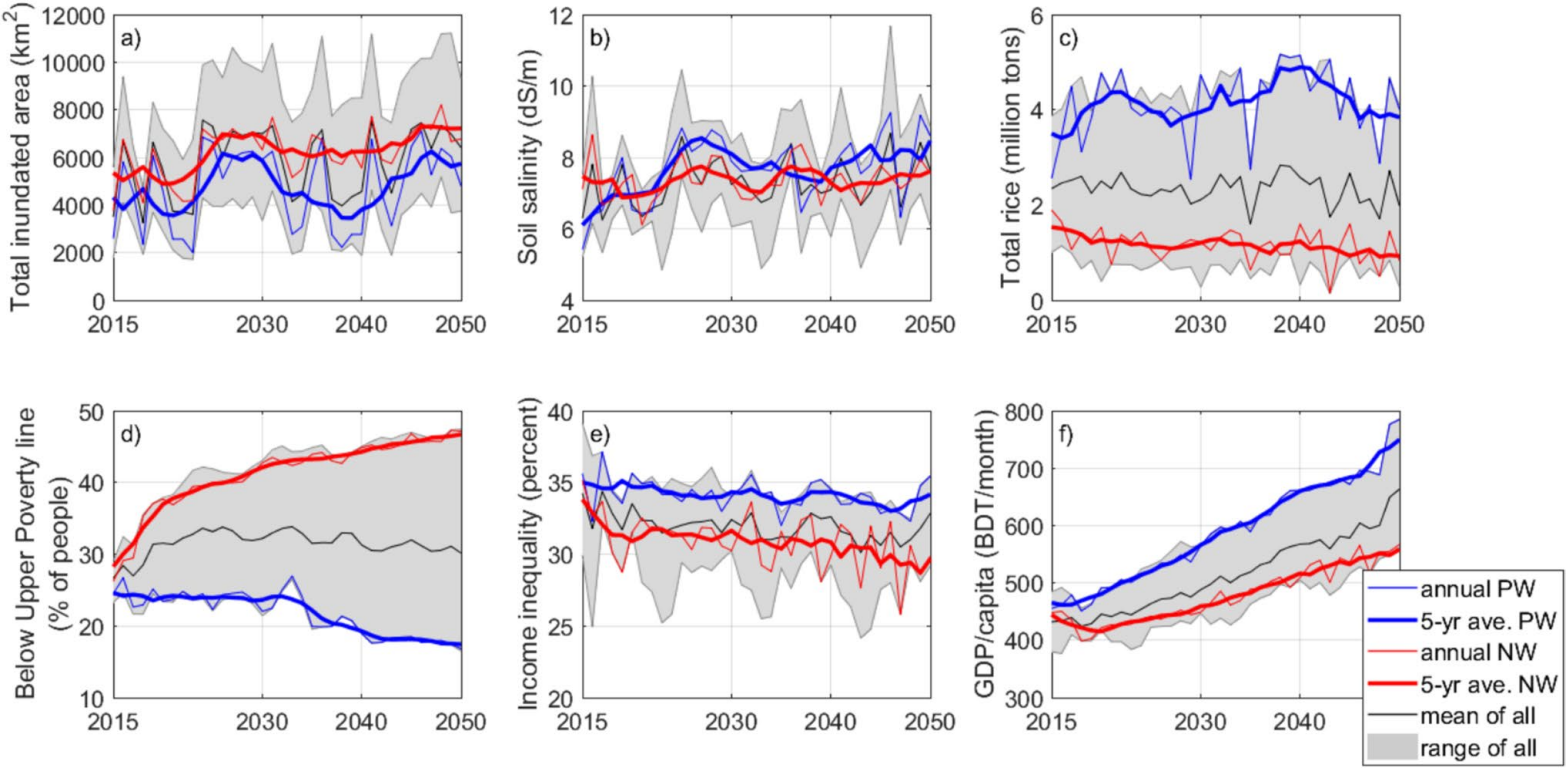
Simulated key indicator trajectories to 2050. (‘PW’ Positive World, ‘NW’ Negative World, ‘all’ means the 36 basic simulations). **a** Inundated area; **b** Soil salinity; **c** Total rice production; **d** Poverty rate; **e** Income inequality (GINI index); **f** GDP/capita. (See definitions in text.) N.B. the Positive and Negative worlds are different in 2015 because the simulation commences earlier (see Methods). For maps in 2050, see Figures S4-S9

*Inundated area* (the maximum annual flooded area due to combined fluvial, tidal and storm surge sources) is simulated to be between 1700 (10% of study area) and 11,200 km^2^/year (57% of study area) with a mean in 2015 and 2050 of 3500 and 6400 km^2^/year, respectively (Fig. 2). Flooding is more severe in the Negative World, while in the Positive World flooding mainly occurs outside the polders (reflecting well-maintained embankments) with high interannual variability and occasional flooding within polders due to extreme cyclones. Flood variability is smaller in the Negative World due to the deterioration of polders, and annual flooding increases towards the maximum possible state.

Maximum annual *soil salinity* (study area average) is between 4 and 12 decisiemens per metre (dS/m, Fig. 2). Sustained soil salinity values of 4 dS/m already limit some crops (e.g. vegetables), and 12 dS/m stops the production of all current crop varieties. On average, soil salinity increases from 6 to 7.6 dS/m by 2050. Soil salinity steadily increases in both the Positive and Negative Worlds due to saline water intrusion to surface- and groundwater sources, and more coastal flooding. However, counterintuitively, modelled soil salinity is slightly higher in the Positive World as (i) there is less monsoon precipitation and thus less flushing of salts from soil by (freshwater) flooding, and (ii) more water-intensive cropping patterns (i.e., more irrigation) leading to higher salt accumulation.

*Total rice production* declines in the Negative World, primarily reflecting increasing flooding and temperature limits being exceeded (Fig. 2). Traditional crop varieties and cultivation methods become progressively less suitable over time. In the Positive World, production increases despite higher soil salinity (reflecting future more resilient crop varieties (BARC 2013)). Short-term falls in annual rice production are attributed mostly to cyclone floods. Spatially, the greatest increases in rice production (Positive World) occur in the areas with higher environmental pressures today (salinity and flooding, Figures S4-S6) because the improvements enable a change from single to multi-season cropping.

*Poverty rate* reflects the cost of basic needs of food and non-food items (known as the Upper Poverty Line)(BBS 2011). The simulated present rural poverty rates (27% for 2015) are comparable with official data for rural areas (35% for 2010) (BBS 2011). In the Positive World, poverty decreases and stabilises around 17% in 2050. In the Negative World, poverty increases to 47%. Note that simulated annual poverty rates were smoothed by coping strategies such as savings, loans or temporary reductions in expenditure, but long-term trends were strongly influenced by scenario assumptions (Lázár et al. 2020a, 2020b).

*Income inequality* slightly decreases in the Positive World to 2050. In the Negative World, income inequality steadily decreases, reflecting that wealthier households experience higher absolute income losses than poorer households, narrowing the income gap.

*GDP/capita* show similar positive long-term economic trends in all cases with a greater increase in the Positive World (Fig. 2). In both Worlds, the off-farm sector dominates economically with a strong positive trend over time irrespective of climate/environmental change. In the Negative World, due to outmigration from the region, the lower population increases per capita land areas, benefiting GDP/capita, which partly offsets the lower off-farm growth rates.

### Sensitivity of key environmental and socio-economic indicators to climatic and socio-economic drivers

We quantified the effect of climatic and socio-economic drivers (Table 1) on the key indicators using a one-at-a-time sensitivity analysis. The mean decadal values were compared with the baseline (2005–2014) value. Figures S11-S16 show the changes in key indicators for the Positive and Negative Worlds for 2050.

We categorised the system sensitivity to the drivers as.

- Low sensitivity if ≤ 25% change in the key indicator.
- Moderate sensitivity if 25 to 50% change in the key indicator.
- High sensitivity if ≥ 50% change in the key indicator.

**Fig. 3:**
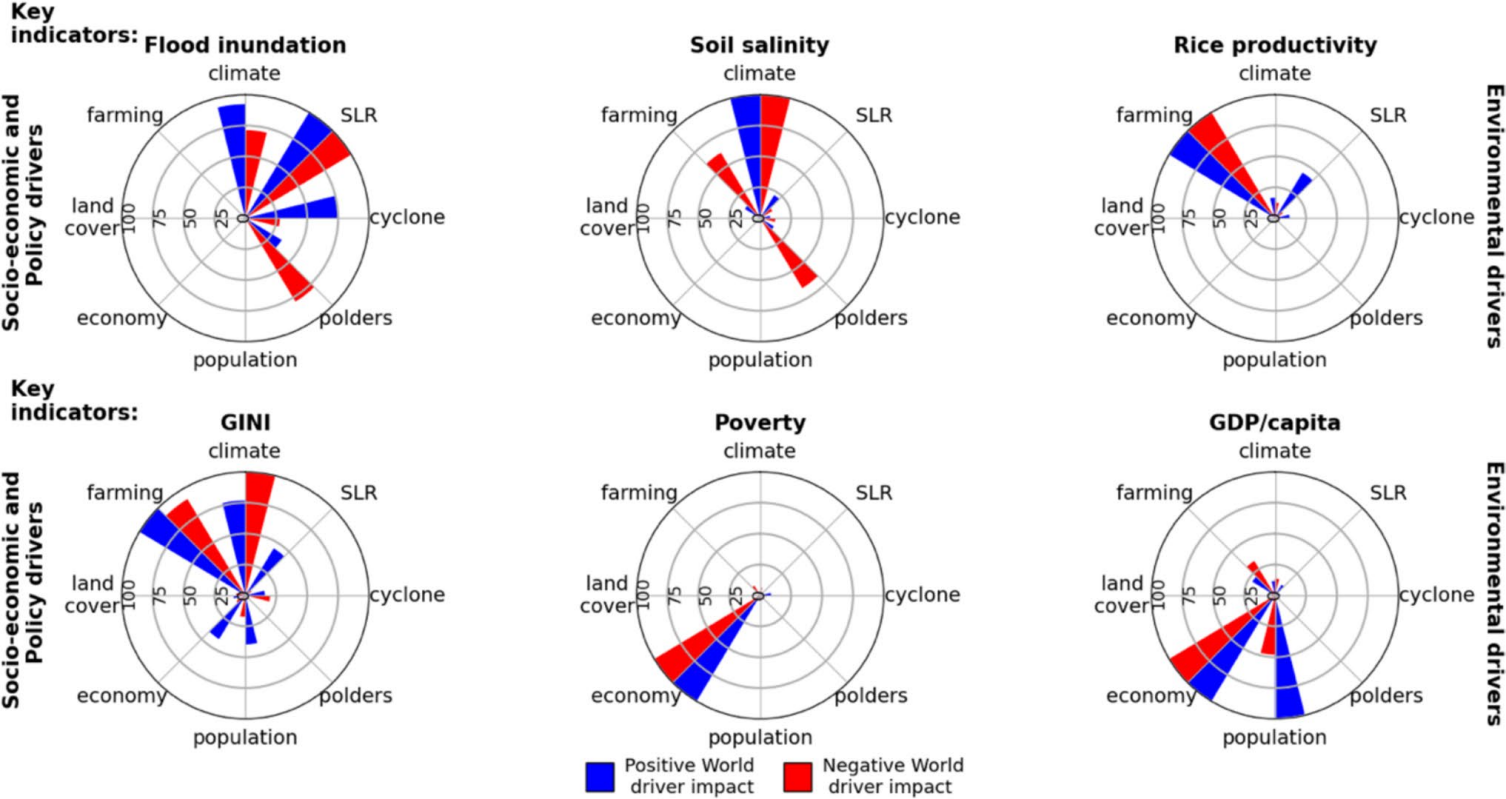
Normalised sensitivity of key output indicators to climatic and socio-economic drivers. The results of Figures S11-S16 are synthesised here by calculating the difference between the minimum and maximum change (i.e. the ‘range’ of change in 2050 compared to the baseline) for every key indicator and driver separately. The length of bars indicates the strength of model sensitivity. (‘climate’: precipitation, temperature and river discharge linked to different climate scenarios; ‘SLR’: relative SLR, ‘cyclones’: cyclones, ‘polder’: embankment maintenance, ‘population’: population size, ‘economy’: economic changes at household level, ‘land cover’: land cover, ‘farming’: farming practices)

The maximum range of the sensitivities (i.e. actual value change) was then calculated for each driver. Normalised values (i.e. actual/maximum) are plotted in Fig. 3.

*Inundated area* is highly sensitive (in descending order) to relative SLR, and to changes in climate-related inputs in both Worlds. Flooding in the Positive World is of high sensitivity to cyclones and in the Negative World it has high sensitivity to polder maintenance. This illustrates that embankment maintenance is a key parameter that should be carefully managed.

*Annual maximum soil salinity* has the highest sensitivity to climate-related inputs in both Worlds, indicating the importance of freshwater flushing of salt from the topsoil. There is high sensitivity to farming practices, in particular irrigation water quality/quantity, and polder maintenance in the Negative World. In the Positive World, salinity is less sensitive to these factors reflecting greater investment in embankments and better crop decisions.

*Rice productivity* is highly sensitive to the crop varieties in use and farm management practices. In addition, relative SLR is moderately important in the Positive World, driving inundation-related damage and soil salinisation.

*Income inequality* is influenced by a variety of drivers. It is highly sensitive to farming practices and climate-related inputs (in both Worlds). This reflects that 85% of households engage in agriculture, so agricultural production influences most households’ incomes. Thus, there might be a widening of income availability between the off-farm, mixed and farming-based households. In the Positive World, inequality outcomes are also moderately impacted by relative SLR (driving flooding), local economy (driving net income) and population size (driving land size and labour availability). Cyclones have a small effect on inequality (everyone in a cyclone path is impacted). In absolute terms, however, the poorest might lose everything, whereas the losses of the less poor are partially offset by their income and savings.

*Poverty rate* is highly sensitive to economic trends alone. This is unsurprising, as market price and fluctuations in household costs directly impact well-being. Although agriculture provides employment for many households in coastal Bangladesh, the steady increase in service sector income buffers the poverty rate to environmental stressors (Figure S3).

*GDP/capita* is highly sensitive to household economies. The Positive World is highly sensitive to population, while the Negative World is moderately sensitive to population and farming practices. Market prices of essential goods, livelihood costs, and potential yields directly impact the net household income. Land size and income levels are controlled by regional population linked to model assumptions on land consolidation. Interestingly, relative SLR, cyclone frequency and polder maintenance have practically no influence on GDP/capita because (i) the rural economy is increasingly dominated by service-based livelihoods and (ii) good farm practices (e.g. more tolerant crop varieties) can compensate for these environmental pressures.

### Influences of climatic changes and adaptation options on population groups and poverty

**Fig. 4:**
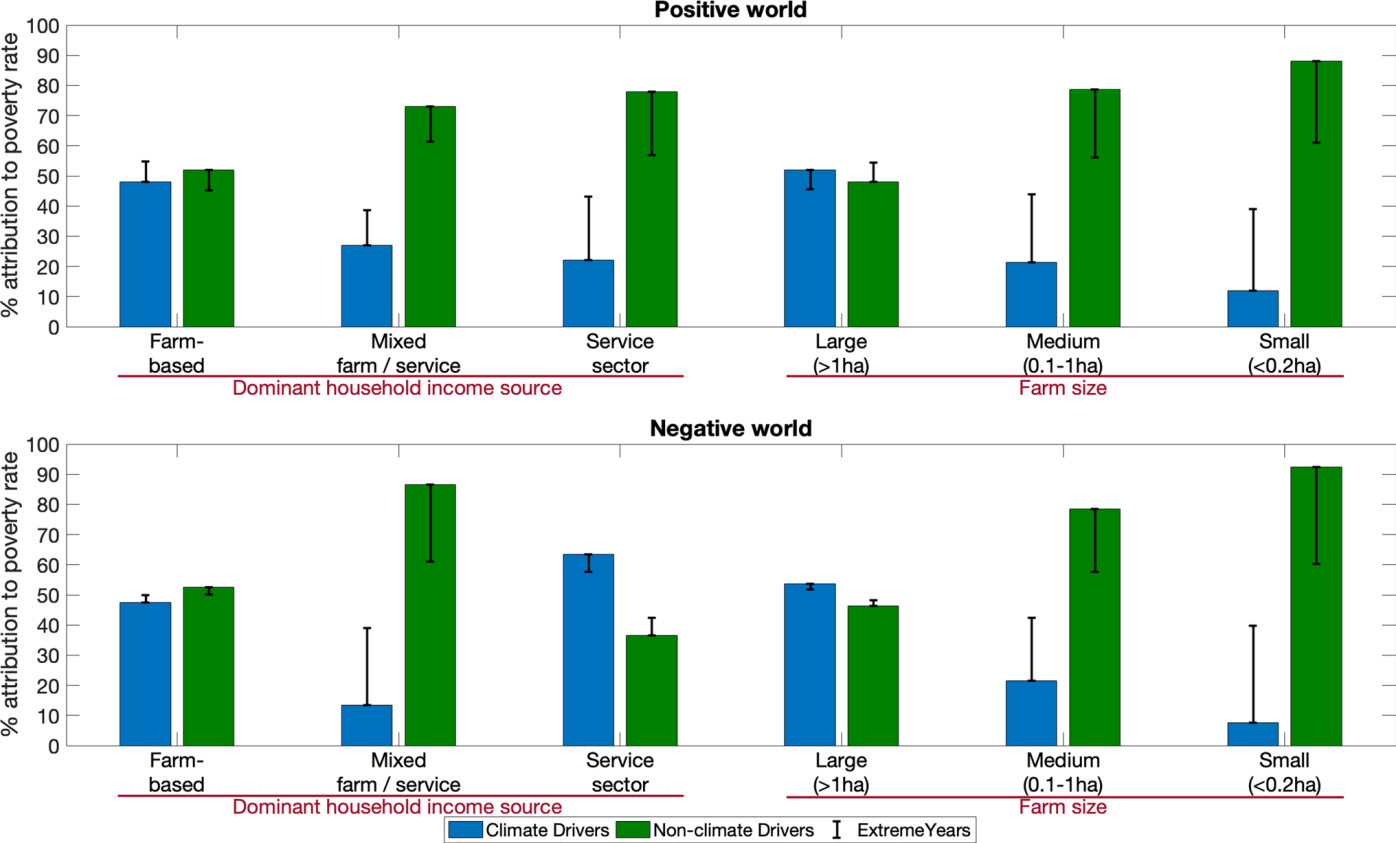
Attribution of climatic and non-climatic drivers to simulated poverty rates of six household types. Bars show decadal mean sensitivity, whereas error lines indicate the same sensitivity in extreme years. The Climate Drivers combine climate-related drivers, SLR and cyclones. Non-climatic Drivers combine embankment maintenance, population size, household economics, land cover and farming practices. ‘Farm-based’ includes all natural resource-based households (farming, fishing, forest collection), where farming is the most dominant livelihood. ‘Mixed farm/service’: households with an equal mix of farm and service sectors. ‘Service sector’: service sector-dominant households

We disaggregated the poverty results by livelihood strategy and land ownership for the Positive and Negative Worlds. We attributed poverty change to climatic drivers by calculating the mean decadal poverty changes for each of these groups due to climatic drivers and compared this to the total decadal poverty changes due to all drivers in percentage terms. The attribution of non-climatic drivers was calculated by subtracting the climatic driver value from 1. Hence, we attributed impacts of climate and non-climate drivers to the poverty rate for differing income groups and land holding sizes (Fig. 4).

In both Worlds, the decadal-mean poverty results are more sensitive to non-climatic drivers. The relative importance of climatic drivers is highly variable, being between 8% and 63% across the household groups (mean, 32% in 2050). In extreme years, poverty attribution to climatic drivers increases significantly compared to the decadal mean (2050: from 32% to 45%). This highlights that climatic drivers (precipitation, temperature, river discharge, cyclones and SLR) are more important in extreme years compared to average years where economic changes mostly drive poverty levels (Figure S15). Thus, more extreme years due to climate change are of concern, and this needs to be further analysed.

Farm-based households have higher sensitivity to climate, and this increases with farm size. Note service sector-dominant households appear as an outlier in the Negative World, where the dominant driver of poverty is ‘climate’. This reflects that the dominantly off-farm households are the most numerous in the study area (~ 70% of all households—see Figure S3), and most large landowners belong to this group. This group owns more climate-sensitive assets (Adams et al. 2016a) resulting in higher climate sensitivity. Extreme year sensitivities are apparent for all groups, reinforcing the importance of farming land as an underlying source of income in the coastal zone.

## Discussion and wider implications

This study highlights that development which sustains well-being and long-term economic activities, including those outside the primary sectors of agriculture and fisheries, need not conflict with climate adaptation. Rather, development can maximise gains while minimising climate losses over the next few decades. This is an important message as the immediate future of coastal Bangladesh, and coastal lowlands globally, is widely portrayed as primarily determined by climate change and SLR (e.g. Huq et al. 1995; Kay et al. 2015; Kulp and Strauss 2019). There is a danger that an overemphasis on climate change as the main determinant of future lowland development can undermine confidence and the willingness to seek sustainable solutions. This can create a self-fulfilling prophesy and potentially suppress development, leading to economic decline and ultimately abandonment (Paprocki 2019). Most contemporary migration decisions are motivated by perceived opportunities (principally towards urban destinations), and facilitated by resource availability to move, rather than current environmental decline (Wrathall et al. 2019; Lázár et al. 2020b; Adger et al. 2021; Bell et al. 2021). Some analyses have suggested the potential for people to keep moving towards the coast despite SLR and climate change because of the benefits provided (Safra de Campos et al. 2020; Bell et al. 2021).

The high sensitivity of the natural environment (e.g. flooding, salinisation) to climatic drivers is apparent in our simulations. However, agricultural productivity is strongly dependent on human actions and investment (Adnan et al. 2020). While inequality, poverty and GDP per capita have weak sensitivities to SLR and cyclone frequencies, they are more sensitive to non-climatic drivers. Nonetheless, extreme climatic conditions do impact livelihoods with variable effects across household types (Becker et al. 2024). Bangladesh has significantly reduced the loss of life during major cyclones, but economic damage remains significant (Needs Assessment Working Group 2020). The threat from extreme climatic events (cyclones, monsoon floods, failed monsoons, etc.) will always exist in Bangladesh and requires ongoing efforts to enhance disaster risk reduction (Lumbroso et al. 2017), including consideration of future climate. Complementary studies on infrastructure provision and investment in Bangladesh support this view (e.g. Adnan et al. 2020; Barbour et al. 2022).

This study demonstrates that development and adaptation have a substantial influence on the present and future delta. We have also shown that climate change is a significant driver of direct and indirect impacts on the economy in coastal Bangladesh. However, our results challenge the idea that climate change adaptation alone is the main priority for Bangladesh. Rather, climate adaptation and development need to be linked and seen as working together to enhance the wealth and resilience of Bangladesh society. We note the so-called ‘Bangladesh paradox’, which highlights sustained and positive health outcomes and relatively high rates of literacy compared to countries with comparable GDP per capita: the paradox is explained by particular configurations of gender equality and strength of civil society (Chowdhury et al. 2013; Hossain et al. 2018; Sultana et al. 2021). Our analysis suggests we should continue to enhance these positive development trajectories. Also, changes beyond 2050 must also be considered, and issues such as accumulating SLR and subsidence must be properly addressed (Becker et al. 2024).

These findings have global relevance (Anthony et al. 2024). Deltas worldwide are experiencing huge pressures, especially from flooding (Edmonds et al. 2020), salinisation (Hassani et al. 2020) and sediment starvation (Dunn et al. 2019). They are vulnerable to climate change, but adaptation, appropriate development and good governance can address the climate impacts at least to 2050 (and possibly longer—Lázár et al. 2020b) and promote resilience and wellbeing of delta societies. However, as we recognise deltas as a system, so this adaptation should also be systemic. One-sided large-scale engineering investments (e.g. embankments) without linkage to other nature-based solutions (e.g. floodplain restoration) and soft adaptation (e.g. investment in human capital) may isolate low-lying land from wider natural processes and thus lock the delta into a cycle of relative SLR (subsidence and global SLR) and progressive embankment raising (Welch et al. 2017; Lázár et al. 2020b; Santos and Dekker 2020). This research supports cross-disciplinary, evidence-based policy formulation to address these issues and demonstrates the IAM approach as a tool to support such analysis. While many of the processes are common (Eslami et al. 2025), each delta would almost certainly require bespoke IAMs tailored to the specific physical, social and policy conditions and issues.

This study reflects multiple years of sustained work and funding in Bangladesh (Nicholls et al. 2018; Rahman et al. 2019). Through stakeholder engagement and technical expertise, we have identified the crucial elements of the coastal Bangladesh system relative to rural livelihoods and built this IAM. While not perfect, it realistically represents and links all major socio-environmental components of the coastal system. Reality is, of course, more complex and more dynamic, and this IAM could be made even more complex, for example, simulating coastal management and household adaptation options dynamically. However, this would require more background studies and resources. This raises the question about limits to the new understanding that such complex IAM can provide and trade-offs in costs and benefits. The general lesson is to recognise the complexity of deltas (and other settings where appropriate) and apply the approaches here as required to develop understanding and evidence concerning future trajectories. This includes considering how human activity might influence them. As such, this research provides a generic framework for such analysis but does not prescribe the details.

It is important to understand that the authors are not saying that development and adaptation can triumph over any change. The magnitude of future climate change and other key factors such as the degree to which basin management is coordinated with upstream countries are critical influences on the future of deltas, especially beyond 2050. Hence, climate mitigation and sustainable catchment scale management are also vital measures (Paszkowski et al. 2021; Becker et al. 2024). However, our analysis identifies specific development and adaptation levers within Bangladesh that can be exploited in the coming decades to reduce the vulnerability and increase the well-being of coastal residents of Bangladesh. This approach is widely transferable.

## Supplementary Information

Below is the link to the electronic supplementary material.

ESM 1 (DOCX 11.2 MB)

ESM 2 (XLSX 45.4 KB)

## Data Availability

All input data are published together with the model code (Lázár et al. 2019), and the data behind the figures are available in the supplementary information files. All raw data generated and analysed during this study are available upon request from the corresponding author.
