# Supplementary material for: Relative importance of climate versus socio-environmental development changes to 2050 in rural coastal Bangladesh—a system analysis
Source: Reg Environ Change. 2026 Feb 7;26(1):38. doi: 10.1007/s10113-025-02511-9 (PMC12881032; doi:10.1007/s10113-025-02511-9)
Supplement: Supplementary file 1 — (DOCX 11.2 MB) [file 10113_2025_2511_MOESM1_ESM.docx]

**Supplementary information**

**Relative importance of climate versus socio-environmental development changes to 2050 in rural coastal Bangladesh – a system analysis**

Attila N. Lázár, Robert J. Nicholls, Craig W. Hutton, Andres Payo, Helen Adams, Anisul Haque, Derek Clarke, Mashfiqus Salehin, Alistair Hunt, Andrew Allan, W. Neil Adger, M. Munsur Rahman, R. Smith

**Table of Contents**

The Delta Dynamic Integrated Emulator Model (ΔDIEM) 3

Figures and Tables providing input information and supporting the manuscript findings 9

References 19

**Figures**

Figure S1: The South-West and South-Central coastal zone of Bangladesh 3

Figure S2: Overview of the Delta Dynamic Integrated Emulator Model (ΔDIEM) showing scenarios/look-up tables and dynamic calculations and the associated model/method 4

Figure S3: Summary of the household livelihood composition of coastal Bangladesh 9

Figure S4: Flood inundation area maps for 2041-2050 (decadal mean, % union area) 9

Figure S5: Soil salinity maps for 2041-2050 (decadal mean, dS/m) 9

Figure S6: Rice production maps for 2041-2050 (decadal mean, tons/union/year) 10

Figure S7: Income inequality maps for 2041-2050 (decadal mean, GINI coefficient - %) 10

Figure S8: Poverty maps for 2041-2050 (decadal mean, Cost of Basic Needs method, Upper poverty line - % population) 10

Figure S9: GDP/capita maps for 2041-2050 (decadal mean, BDT/month) 11

Figure S10: Total population maps for 2041-2050 (decadal mean, thousand) 11

Figure S11: Sensitivity of the inundated area output to different drivers within the Positive and Negative world scenarios at mid-Century 11

Figure S12: Sensitivity of the soil salinity output to different drivers within the Positive and Negative world scenarios at mid-Century 12

Figure S13: Sensitivity of the rice productivity output to different drivers within the Positive and Negative world scenarios at mid-Century 12

Figure S14: Sensitivity of the income inequality (GINI coefficient) output to different drivers within the Positive and Negative world scenarios at mid-Century 12

Figure S15: Sensitivity of the Cost of Basic Needs (Upper Poverty Line)-based poverty rate output to different drivers within the Positive and Negative world scenarios at mid-Century 13

Figure S16: Sensitivity of the GDP/capita output to different drivers within the Positive and Negative world scenarios at mid-Century 13

**Tables**

Table S1: Details of the input scenarios 14

Table S2: List of randomly selected, simulated cyclones 17


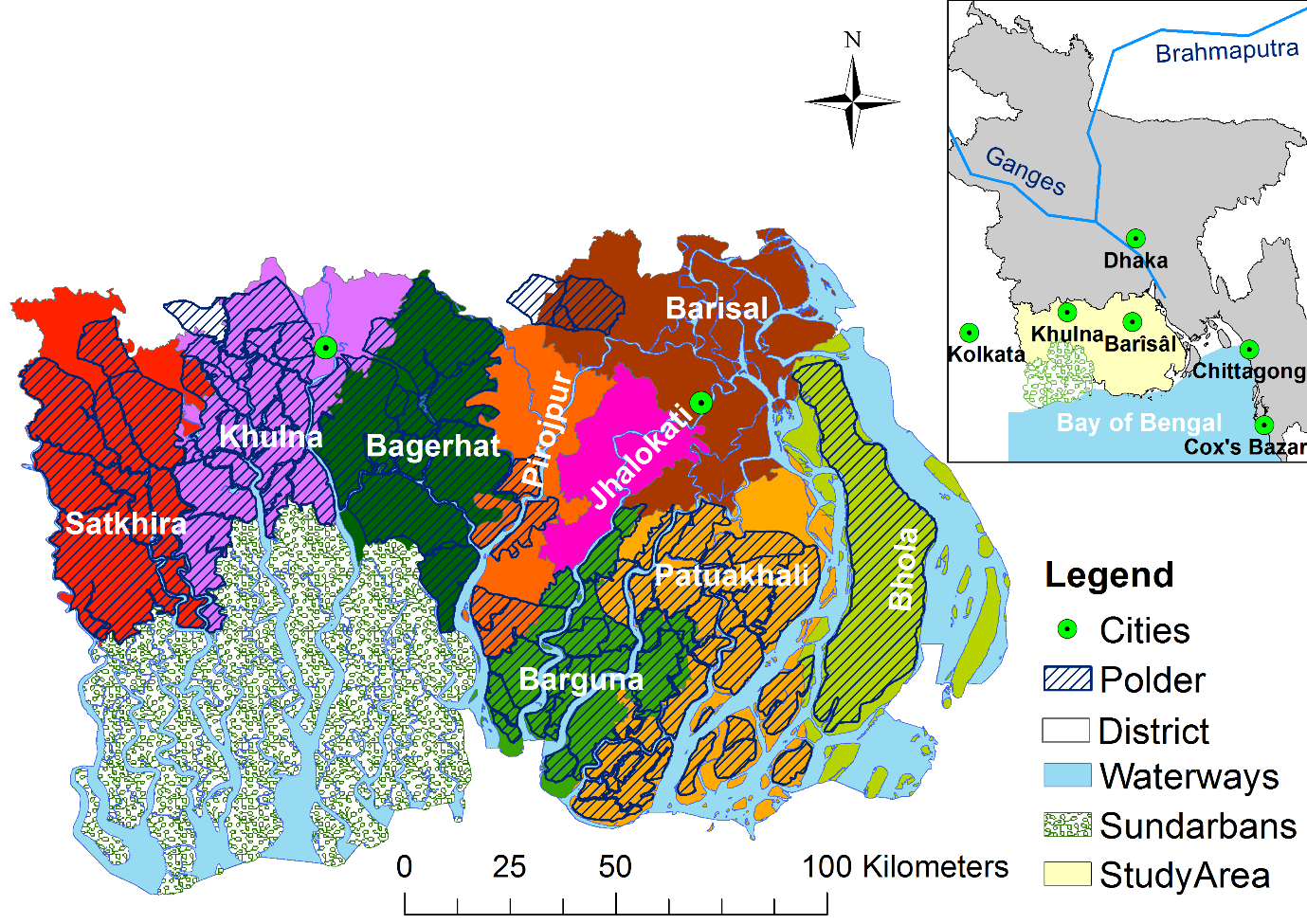


**Figure S1**: The South-West and South-Central coastal zone of Bangladesh highlighting the districts and polders.

***The Delta Dynamic Integrated Emulator Model (ΔDIEM)***

ΔDIEM is a trans-disciplinary Integrated Assessment Model (IAM) framework designed to analyse linkages between natural resources, livelihoods, well-being and governance in coastal Bangladesh. This is a novel, tightly coupled integrated assessment platform capturing a range of biophysical (climate, upstream hydrology, sea elevation, fisheries) and socio-economic (demography, economy, land use, household coping behaviours) drivers and factors and designed to support system-level delta analysis and planning in Bangladesh (Nicholls et al. 2016, Hutton et al. 2018, Lázár et al. 2018) (Figure S1). ΔDIEM encapsulates these drivers to assess the cumulative effect of changes on the well-being and poverty of the coastal population (Lázár et al. 2020). ΔDIEM outputs include calculated flooded area, soil salinisation, agriculture productivity, livelihood potential and human well-being, as well as income inequality, poverty, and GDP, disaggregated by sub-populations. Thus, ΔDIEM offers several advantages compared to earlier assessments by including: (i) climate, environmental and socio-economic drivers, (ii) interactions between model components including scale harmonisation, (iii) coastal management and governance dimensions, and (iv) household coping and adaptation at the local level for multiple archetypal household types.


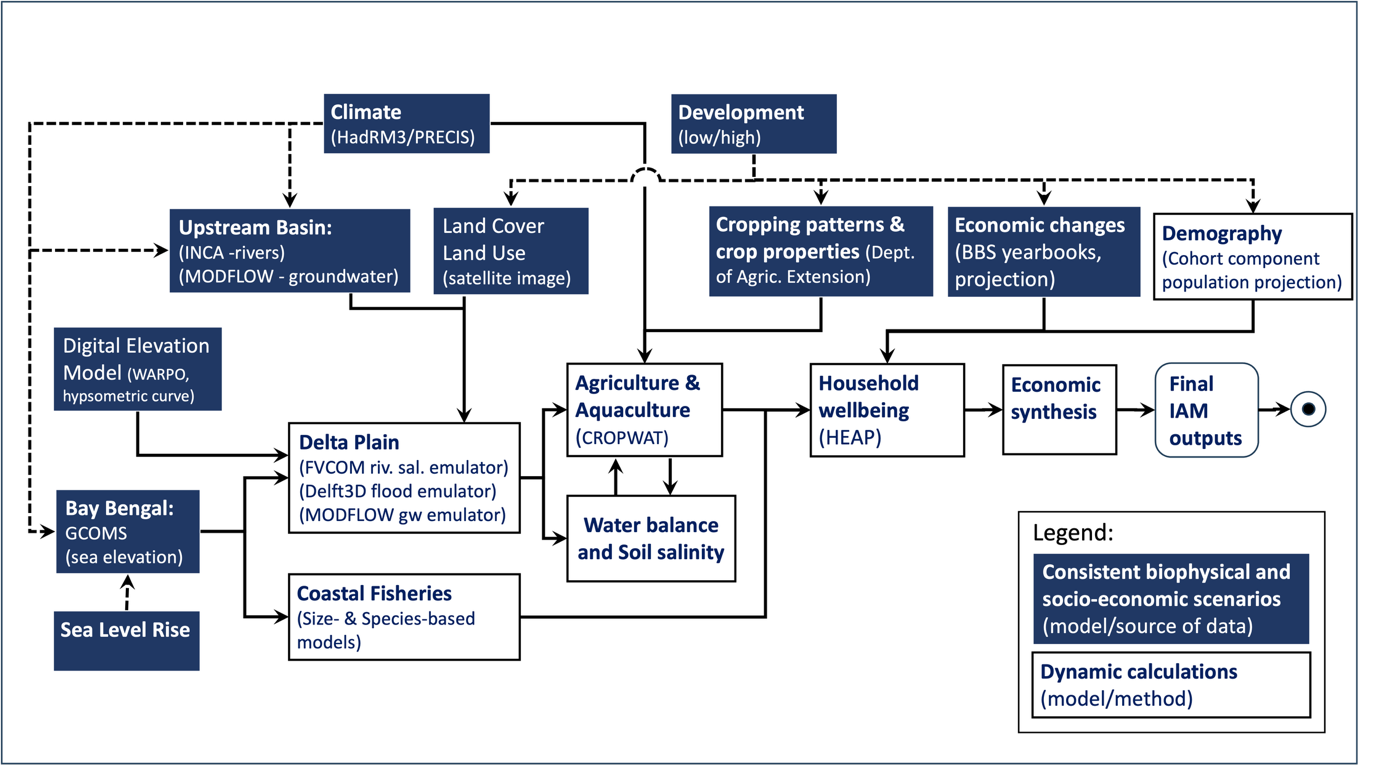


*Figure S2: Overview of the Delta Dynamic Integrated Emulator Model (ΔDIEM) showing consistent biophysical and socio-economic scenarios in look-up tables based on libraries of model simulations or published data, and dynamic calculations and the associated model/method.*

ΔDIEM and its scenario framework were developed via sustained engagement with diverse Bangladeshi stakeholders at multiple levels of influence, ranging from national Ministries and development agencies to local NGOs and academic experts (Nicholls et al. 2018, Allan et al. 2022). ΔDIEM allows the testing of a large number of water-based structural and policy interventions (e.g. embankment changes, new crops, subsidies, loan structures, etc.) within a robust scenario framework, as well as quantify different development trajectories and their trade-offs (Hutton et al. 2018, Rahman et al. 2019).

The simulation period in this paper is 1985-2050, where the 1985-2004 period is the spin-up period, the 2005-2014 period is the baseline and the analysis focuses on the 2015-2050 period. ΔDIEM produces results for 653 ‘Unions’, which are the smallest administrative unit in Bangladesh. On average, each Union comprises up to 9 villages with ~21,000 people over 26 km^2^. Note that the city of Khulna and Barisal with 1 million inhabitants in total are excluded from our analysis given their urban nature.

Drivers and processes outside the coastal zone are part of the boundary conditions of the model, meaning that they are user-specified input scenarios (Figure S2). These include complete, transient climate (Caesar et al. 2015), upstream hydrology (Whitehead et al. 2015), Bay of Bengal sea elevation and fishery (Fernandes et al. 2016), demography (Szabo et al. 2016) and economic (Hunt 2018) time series for the period of 1981-2050. We calculated relative sea-level rise (SLR) (Table 1) by combining the climate-induced SLR (26cm or 61cm in 2050) with a uniform linear subsidence of 2.5mm/year. Land use and land cover are also input scenarios in this version of the model that are based on the remote sensing analysis of historical trends (Amoako Johnson et al. 2016, Mukhopadhyay et al. 2018) and stakeholder narratives about the future (Allan et al. 2022). These inputs are look-up tables from which the appropriate time series is selected during the model run. However, users are allowed to customise the population projections through modifying the cohort component population projection method inputs (life expectancy, fertility rate, life tables, migration rates), the FarakkaTreaty (1996) which influences water sharing of the Ganges flow between India and Bangladesh, and the future economic assumptions.

The biophysical aspects of the coastal zone are captured dynamically with both statistical emulators and process-based calculations (Figure S2). Representation of coastal hydrology is based on a library of model runs of detailed three-dimensional, physics-based models such as Delft-3D (surface water quantity), FVCOM (river salinity) and MODFLOW-SEAWAT (groundwater quantity and quality). Such models are computationally too expensive to run continuous multi-decadal simulations within an IAM, but they are physics-based and capture the coastal processes realistically. ΔDIEM uses statistical emulators (Payo et al. 2017) to simplify these complex model results in a way that allows fully coupled, continuous and integrated model simulations. The emulators combine the Partial Least Square regression with Canonical Correlation Analysis to represent the simulated spatial and temporal dynamics of these complex models by statistically establishing a relationship between inputs (e.g. incoming river discharge, daily sea elevation statistics) and outputs (e.g. river elevation, inundated area, mean inundation depth). The training and predictions use Equation 1:

$Y_{n*q}=X_{n*p}* \Psi_{p*q}$ [1]

where Y - a matrix of the outputs (e.g. flood extent; n - daily simulation results, q – simulation units, i.e. Unions), X – a matrix of predictors (n - daily simulation results, p – predictors, e.g. sea-level height, river discharge, etc), The latent variable (i.e. Ψ - Empirical Orthogonal Eigenfunctions) is calculated as:

$\Psi=A*\Lambda*B^{T}*S$ [2]

where A and B are canonical coefficients, Λ is the diagonal square matrix, and S is the covariance matrix of Y (**_
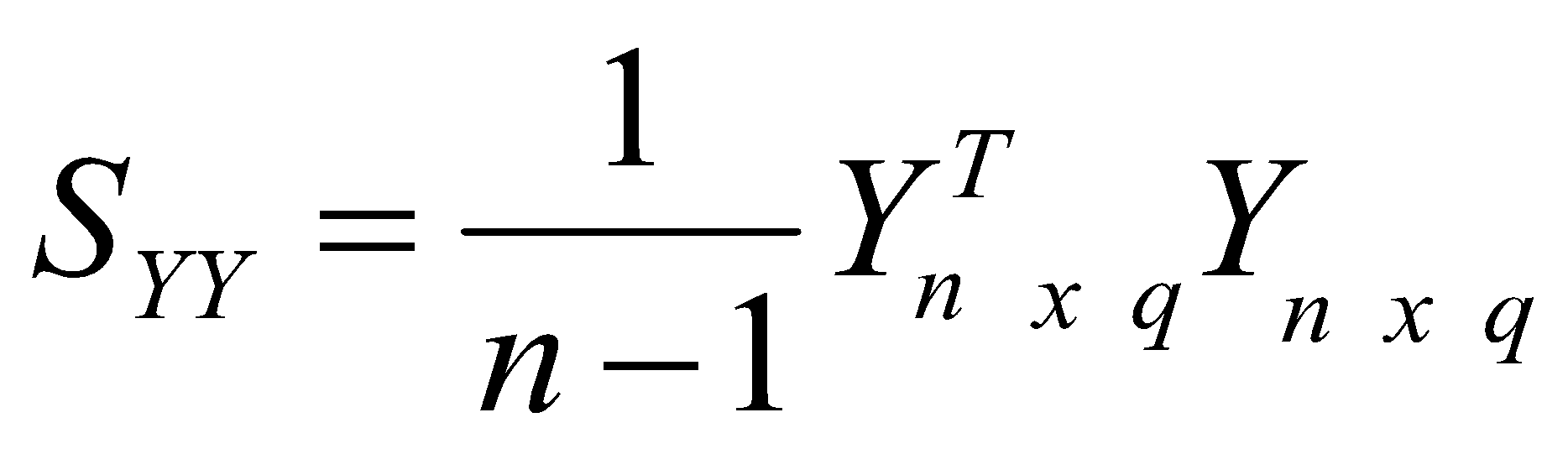
_**).

This modelling technique is useful when the focus of the analysis is not on the actual mechanics of changes, but rather the changes in the outputs. Emulators are computationally inexpensive allowing a daily emulation over one hundred years within just a few seconds; whereas, for example, Delft-3D requires approximately 24 hours for a one year simulation on a PC.

ΔDIEM represents two biophysical issues with greater detail: soil salinization and farming practices, because agriculture underpins rural livelihoods and the economy and thus warrants more detailed calculations and scenario runs. The soil salinization process is complex with drivers of precipitation, evapotranspiration, capillary rise, infiltration, deep percolation, surface water drainage, flooding and irrigation. ΔDIEM fully couples these drivers in a water and salt-balance calculation (Payo et al. 2017). The soil water content is governed by the porosity, the depth of root zone (plant growth cycle-driven), and the water inputs and discharges. The water balance equation is similar to the salt-mass balance equation:

$y_{rz,t}=y_{r,z,t-1}+IC_{i}+GC_{g}+IrrC_{irr}-RC_{rz}$ [3]

where y is the salt mass content in (gr) at the root zone (i.e. ‘rz’) at a given time (t), C – salt concentration of infiltrated water (i), groundwater (g), irrigation water (irr) and root zone (rz); I – infiltration, G – capillary rise, Irr – irrigation, R – groundwater drainage. Infiltration, percolation, capillary rise, groundwater discharge are calculated by ‘standard’ hydrological equations, but the surface water drainage uses a simplified formula:

${DR}_{smax,t}=f_{R}{DR}_{max}$ [4]

where 𝐷𝑅𝑠𝑚𝑎𝑥 is the maximum surface drainage rate, 𝑓_𝑅_ - drainage correction factor that is linked to river elevation (i.e. high water level reduces the hydraulic gradient of surface waterflow). ΔDIEM works at Union-scale, and detailed drainage information is not available, thus the maximum drainage rate is approximated from long-term average monsoon rainfall totals and from waterlogging information (i.e. by what date would the ponding monsoon water disappear from the soil surface – using unpublished upazila soil characteristics data from the Bangladesh Department of Agricultural Extension, DAE).

Farming is the most important natural resource-based livelihood in coastal Bangladesh. Therefore, ΔDIEM represents this with a process-based model: the FAO’s CROPWAT model simulating crop development based on available water (Allen et al. 1998). In ΔDIEM, however, this agriculture model is extended with salt-, temperature- and flood-water related limitations and atmospheric fertigation to be able to capture climatic, environmental and human drivers of change (Lázár et al. 2015). The extended CROPWAT model is also able to consider both traditional agriculture and pond-based aquaculture crops, allowing representation of integrated farming settings. The extended CROPWAT model, and thus the simulated cropping patterns and irrigation water use are fully coupled with the soil salinity calculation (Payo et al. 2017) because irrigation water quality can be a major driver of salinity build up (Clarke et al. 2015).

ΔDIEM links biophysical changes to livelihood potential and simulates household well-being with a process-based, agent-based-type calculation routine (Lázár et al. 2020). This model was built on census data and a novel household survey (Adams et al. 2016a, Adams et al. 2016b) and simulates the well-being trajectories of 36 archetypal households (i.e. household types). The household types are developed based on observed seasonal variations of the six occupation types: (i) farming (agriculture/aquaculture/farm animals), (ii) farm labour, (iii) fishing, (iv) forest goods collection, (v) manufacturing, and (vi) small business activities. ΔDIEM calculates farming, farm labour requirement and fish catches dynamically. Forest goods collection is a static input as the *Sundarbans* mangrove forest is highly unlikely to disappear over the time span of the analysis (Payo et al. 2016) and its basic services (e.g. timber, fruits, honey, flood protection) are available in all mangrove species assemblages as long as the forest is present and accessible (Mukhopadhyay et al. 2015). Finally, non-natural resource-based livelihoods (businesses, services and manufacturing) were outside the scope of the research. Thus, these are simply represented as an input scenario (Hunt 2018).

The multidimensional well-being of the households is expressed through a set of simulated expenditure levels. Expenditure levels are used as poverty indicators over income or assets, because they indicate if basic needs are met and clearly indicate if the households can survive seasonal poverty using coping mechanisms (Falkingham and Namazie 2002). The household expenditure is estimated from an income-expenditure mass-balance calculation:

*Residual Income = Total Income + Total Savings – Fixed Expenditures =*

*= Total Livelihood Income + Total Savings + Remittances +*

*Loan Income – Livelihood expenditures – Loan expenditure* [5]

The calculation uses an optimisation routine that matches the income and fixed livelihood costs to the affordable level for food, essential and non-essential house items, education, health and other expenses for the specific archetypal household (Lázár et al. 2020). The affordable expenditure is calculated


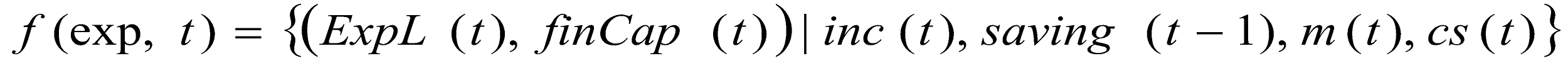
 [6]

where the expenditure level *exp* (a set of five expenditure levels), *ExpL* is the current *expenditure level, finCap* is the financial capacity level (a set of six levels), *inc* is the income, *savings* is the total value of the cash and non-productive asset savings, the number of labouring members of the household *m*, and cs is the selected coping strategy (a set of five see below). The problem is to find a solution that: (1) minimises the number of members engaging in labour work (i.e. maximising the deviation between the total members and the labouring members), (2) minimises the number of coping strategies (i.e. maximising the deviation), but (3) maximises the financial capacity level while maintaining a minimum net savings (i.e. at least 10% of income remains after paying all expenditures and expenses):

$max\sum_{m} \sum_{cs} \left\{ 0.9*inc\left( t \right)-f\left( exp,t \right),0 \right\}$ [7]

where *m* is the number of members engaging in labouring activities, *cs* is the applied number of coping strategies, *inc* is the total income, *exp* is the total expenditure, *t* is timestep ‘t’.

This optimisation includes five coping mechanisms (cash savings, selling assets, loans, reduced expenditure, temporary agriculture labouring) in addition to two safety nets: postponing loan repayment and support from friends and family. Furthermore, more intense engagement in farm labour is an additional coping strategy for the simulated households in stressed economic situations. Food expenditure roughly correlates with health through calorie and protein intake. Each simulated food expenditure level contains a certain amount of calories and proteins that are based on the Household income and Expenditure Survey of Bangladesh (BBS 1991) and the ESPA Deltas household survey observations (Adams et al. 2016b). Finally, by knowing the monthly household economics and calorie intake levels, food security indicators, such as hungry periods, and other monetary poverty indicators, such as GINI coefficient (i.e. income inequality) and GDP/capita, can be easily calculated at different scales (household/district/delta).

ΔDIEM handles hydrology, flooding, salinization and farming with a daily timestep and at the Union-scale, but these calculations are done separately for protected (within polders) and non-protected areas (outside polders). Demography is simulated with a 5-year timestep and at district scale that is then downscaled to Union-scale and to monthly timestep using Census data (BBS et al. 2012). The household well-being and poverty calculations are done for each household type at the scale of the Union and with a monthly timestep. Protected and non-protected households are separately calculated.

ΔDIEM is coded and run in Matlab version 2017a (MathWorks 2017). ΔDIEM and its documentation is publicly available (Lázár et al. 2019).

The inputs used in the simulations analysed in this paper are provided in Table S1. Detailed mapped results for the key simulated indicators shown in Figure 2 for the period 2041-2050 are given in Figures S4 to S9. Figures S11 to S16 give the corresponding sensitivity analyses which inform Figure 3. Figure S10 provides the population distribution for the period 2041-2050.

**Model limitations**

The ΔDIEM modelling approach is detailed and complex, but it still has important limitations that are beyond the current scope of the project. The model simulates observed farming practices, but crop changes due to weather conditions are not simulated. There is no household adaptation beyond coping, and thus households react but do not make long-term predictions and significant livelihood changes. To address these, further field observations and empirical insights would be required together with the development of a more detailed agent-based model. The micro-economic changes are based on expert elicited time series and these are not dynamically linked to environmental and demographic changes in the simulations. Selling and buying of land is not simulated and landholdings are equiproportionally distributed based on aggregate demographics and land cover changes in a Union. Finally, household behaviour and economic trend projections are very uncertain beyond 10-20 years into the future which is why we stop simulations and analysis at 2050. Hence, this emphasises that the integrated assessment results have to be treated as a sensitivity and trend analysis, rather than providing realistic projections. IAMs of this type are still unusual and all have made trade-offs on system representation and output scale. Thus there is significant potential for further development and improvement of both the model framework and the detailed implementation to address new questions, or the approach being operationalised into management practise.

**Validation**

Validation is the process of establishing confidence in the soundness and usefulness of a model (Forrester and Senge 1980). Validation begins as the model builder accumulates confidence that a model behaves plausibly and generates problem symptoms or modes of behaviour seen in the real system. Confidence in system dynamic models can be increased by a wide variety of tests that include tests of model structure, model behaviour and model’s policy implications. The tests used to build confidence on ΔDIEM comprised three boundary adequacy tests: (1) model structure verification to check whether or not model aggregation is appropriate and if the model includes all relevant structure, (2) behaviour reproduction test to check how well model generated behaviour matches observed behaviour in real systems (i.e. testing each component separately against observations and testing the full model against observations), and (3) behaviour sensitivity test focused on sensitivity of model behaviour to changes in parameter values and policy interventions. Since these testing and validation results are reported in full in the above publications, we only briefly summarise them here.

The coastal hydrological emulators on river elevation, river salinity, depth to groundwater, groundwater salinity, inundation area and mean inundation depth are in good agreement with the detailed, high fidelity models of Delft-3D, FVCOM and MODFLOW-SEAWAT (Payo et al. 2017). The largest errors occur at the lower values and the smallest errors occur at the highest values. Thus, for example, large inundation areas have lower associated errors, whereas small inundation areas have higher emulation uncertainties. This is fortunate, because larger events (inundation, salinisation) are hydrologically the most important. The Root Mean Square Error (RMSE) of the emulators are 0.012-0.13 m for inundation depth, 1.36 ppt for river salinity, 0.35 m for river elevation, 0.1m for depth to groundwater and 0.072 ppt for groundwater salinity.

Soil salinity simulation results were evaluated against Dasgupta, et al.(Dasgupta et al. 2015) for 2001 and 2009. Annual median error is 2.1-2.6 dS/m, the dry season maximum salinity error is 2.6-3.6 dS/m and for the wet season it is 2.9-3.5 dS/m. The largest errors were caused by the observed moderately high dry and wet season soil salinities (4-16 and 4-12 dS/m, respectively) in the northern part of Khulna and Bagerhat districts. The simulated ΔDIEM soil salinities were very low, because the FVCOM and MODEFLOW-SEAWAT models estimate low salinities for both river and groundwater (0-1 ppt).

Crop simulations were compared with observations from nine districts and nine sub-districts and the fit was good both spatially and temporally for the 2000-2010 period (Lázár et al. 2015). The RMSE was 2.3-11.9% for all the major, important crops (e.g. rice varieties, chilli, grass pea). Less important crops of coastal Bangladesh, such as wheat and potato, were simulated with less accuracy (RMSE: 22-70%).

The household well-being outputs were evaluated against observations on total expenditure, calorie intake, protein intake, GINI coefficient and 1.90 USD/capita/day headcount observations available from HIES2010 and World Bank datasets (Lázár et al. 2020). The errors were not quantified because the observations were only available at national or regional level at best. However, the simulated values captured the magnitude and trend of the observations well, thus increasing the confidence in the overall behaviour of the full ΔDIEM model.

***Figures and Tables providing input information and supporting the manuscript findings***

**
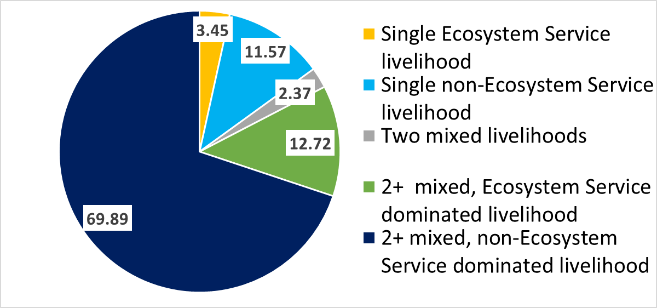
**

*Figure S3: Summary of the household livelihood composition of coastal Bangladesh. Total number of interviewed households: 1478 (consistent across all three seasons). Data source: the ESPA Deltas household survey (Adams et al. 2016a).*


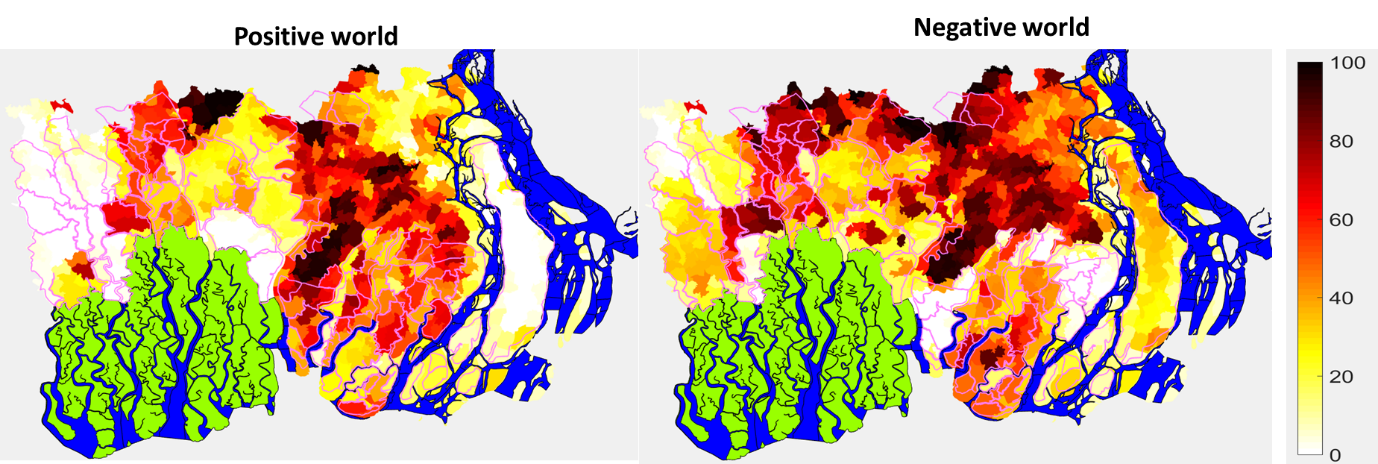


*Figure S4: Flood inundation area maps for 2041-2050 (decadal mean, % union area). Shaded colours show the output variable value. Purple lines show the polder boundaries. Green areas are the Sundarbans forest. Blue areas are significant river channels.*


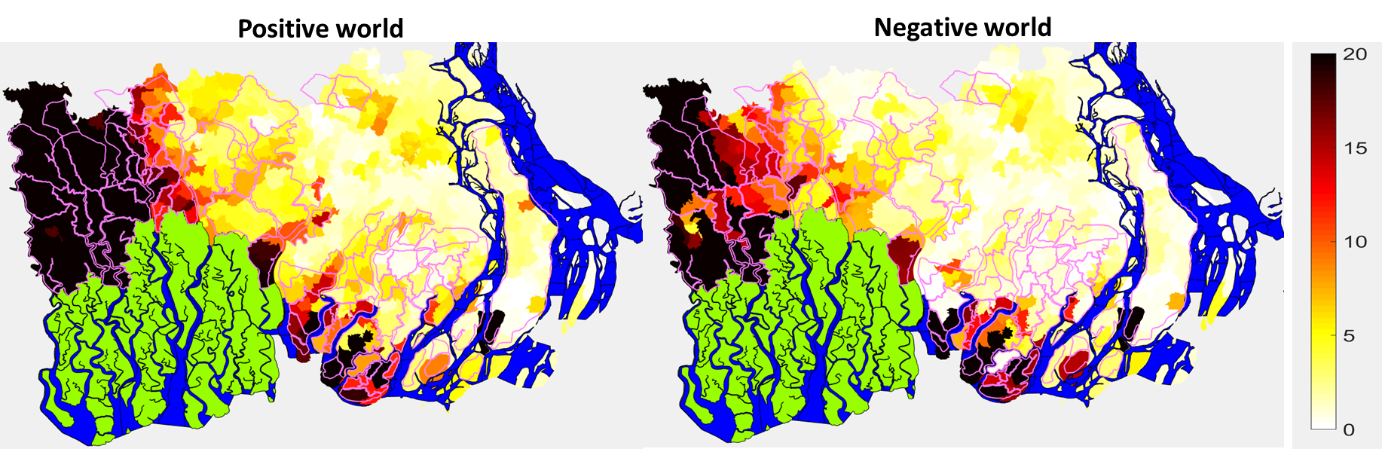


*Figure S5: Soil salinity maps for 2041-2050 (decadal mean, dS/m). Shaded colours show the output variable value. Purple lines show the polder boundaries. Green areas are the Sundarbans forest. Blue areas are significant river channels.*


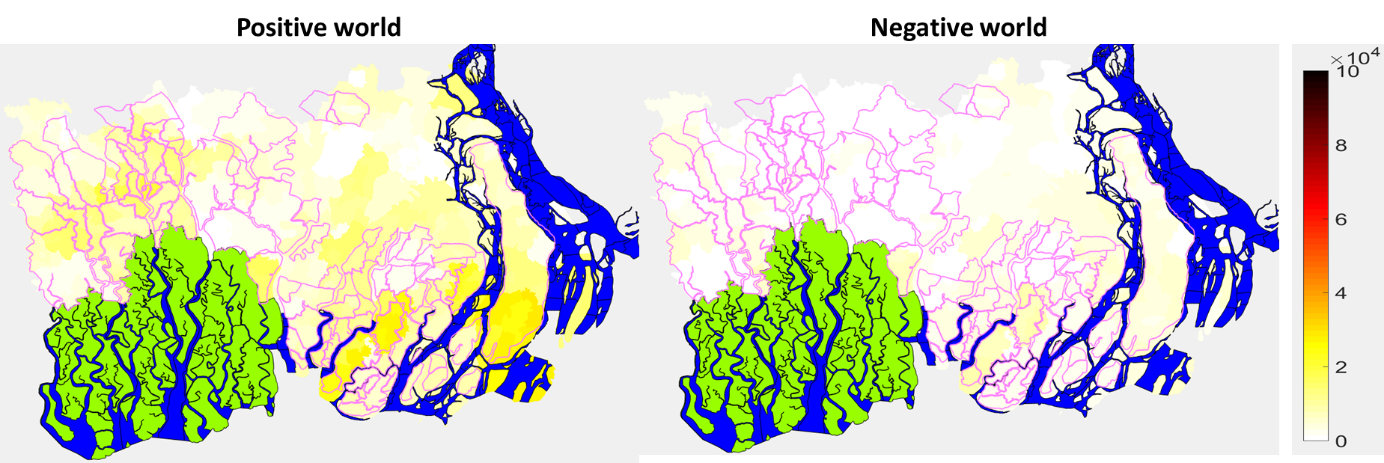


*Figure S6: Rice production maps for 2041-2050 (decadal mean, tons/union/year). Shaded colours show the output variable value. Purple lines show the polder boundaries. Green areas are the Sundarbans forest. Blue areas are significant river channels.*


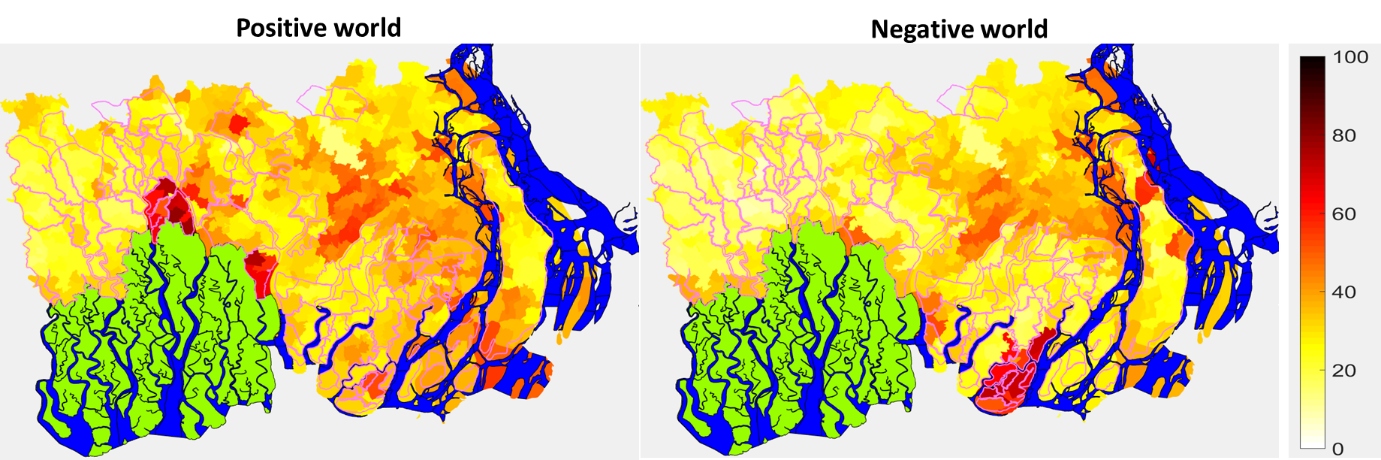


*Figure S7: Income inequality maps for 2041-2050 (decadal mean, GINI coefficient - %). Shaded colours show the output variable value. Purple lines show the polder boundaries. Green areas are the Sundarbans forest. Blue areas are significant river channels.*

*
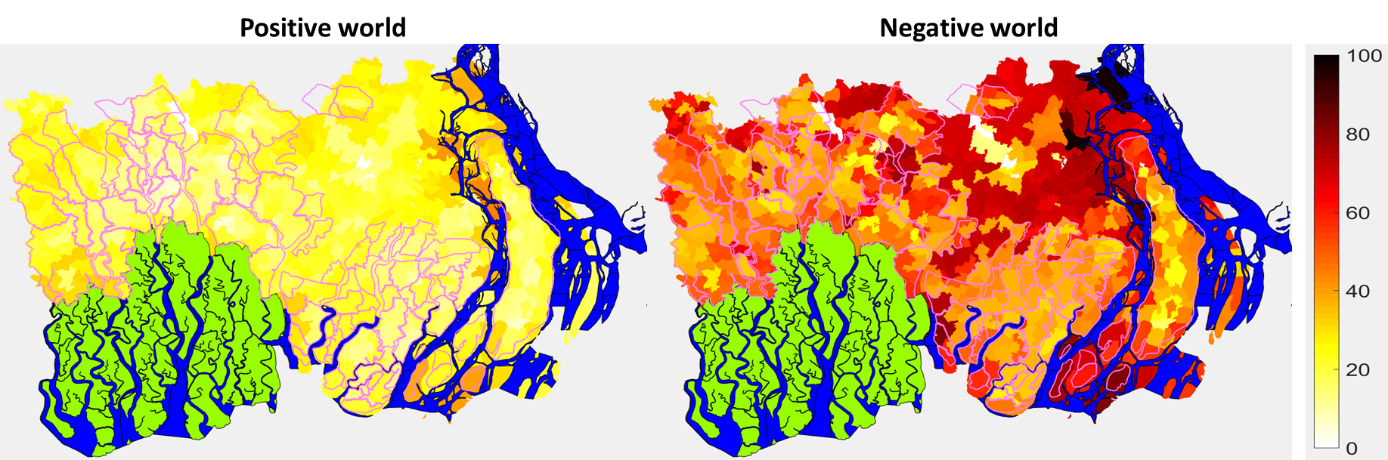
*

*Figure S8: Poverty maps for 2041-2050 (decadal mean, Cost of Basic Needs method, Upper poverty line - % population). Shaded colours show the output variable value. Purple lines show the polder boundaries. Green areas are the Sundarbans forest. Blue areas are significant river channels.*


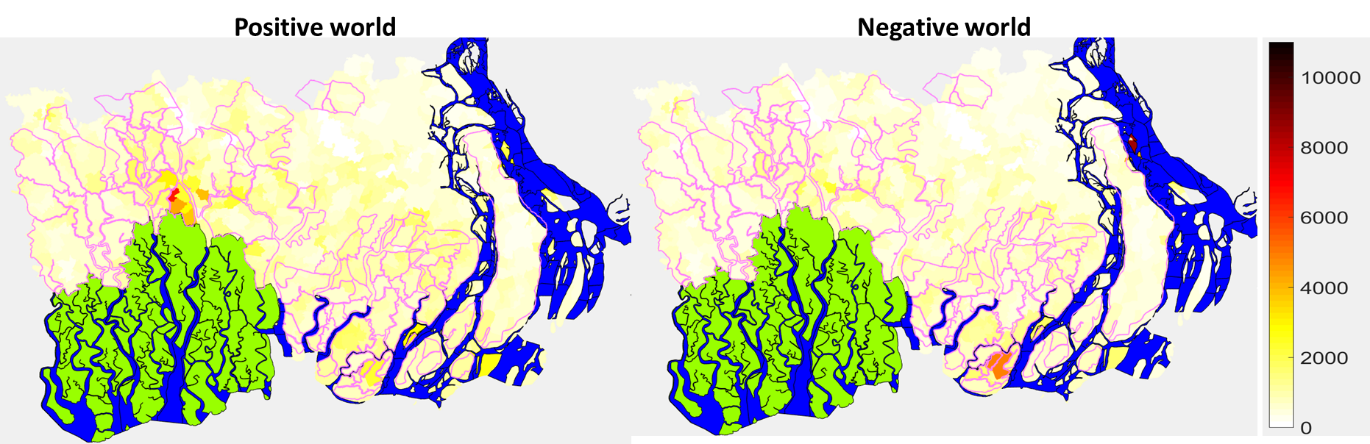


*Figure S9: GDP/capita maps for 2041-2050 (decadal mean, BDT/month). Shaded colours show the output variable value. Purple lines show the polder boundaries. Green areas are the Sundarbans forest. Blue areas are significant river channels.*


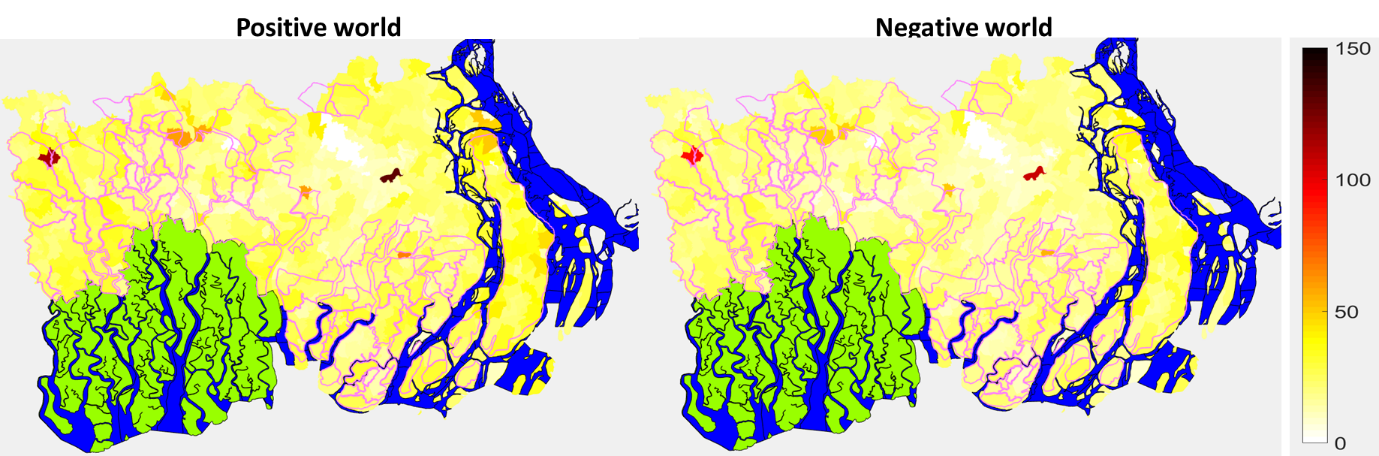


*Figure S10: Total population maps for 2041-2050 (decadal mean, thousand). Please note that population of large cities (Barisal and Khulna) are not shown. Shaded colours show the output variable value. Purple lines show the polder boundaries. Green areas are the Sundarbans forest. Blue areas are significant river channels.*


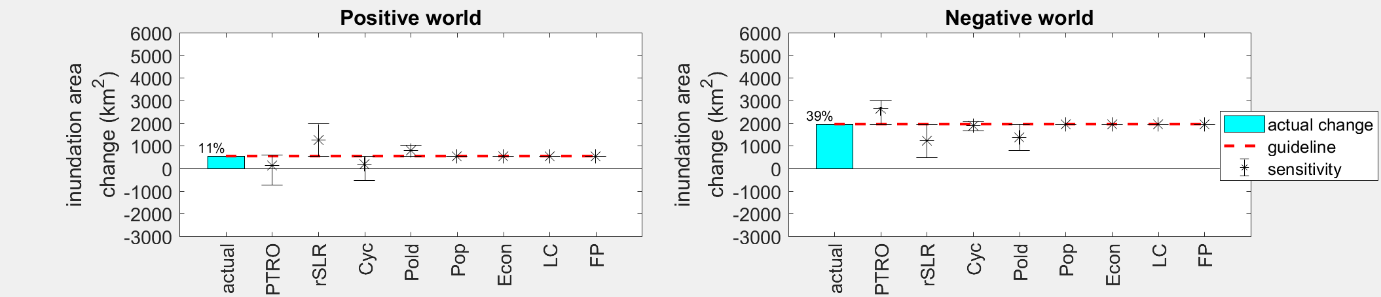


*Figure S11: Sensitivity of the inundated area output to different drivers within the Positive and Negative world scenarios at mid-Century. The calculation compares the baseline results (2005-15) with the mid-century (2041-50) results. The decadal (annual) minimum, mean and maximum values were calculated first for the two periods. The* ***‘actual change’*** *blue bar shows the expected mean decadal change in inundated area compared to the baseline (km^2^, also expressed as a percentage value: e.g. +13% increase compared to baseline). The* ***red line*** *enables the visual comparison of the blue bar with the error bars. The* ***error bars*** *show the change in the decadal minimum, mean and maximum as a result of changing just one driver, e.g. the sea level rise, and keeping all other drivers constant. (Abbreviations: ‘actual’: decadal mean change compared to baseline, ‘PTRO’: precipitation, temperature and runoff linked to different climate scenarios, ‘rSLR’: relative sea level rise, ‘Cyc’: cyclone frequency, ‘Pold’: polder maintenance, ‘Pop’: population size, ‘Econ’: economic changes at household level such as selling price of crops, cost of food, etc., ‘LC’: land cover, ‘FP’: farming practices)*


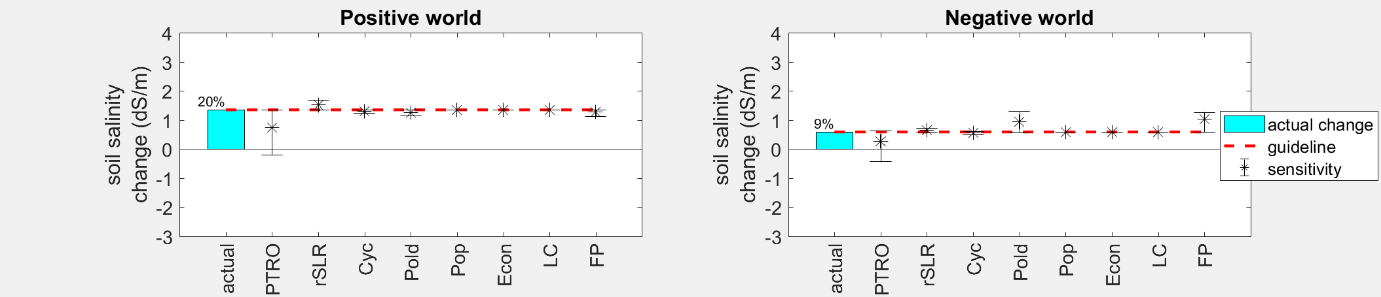


*Figure S12: Sensitivity of the soil salinity output to different drivers within the Positive and Negative world scenarios at mid-Century. The* ***‘actual change’*** *blue bar shows the expected mean decadal change in inundated area compared to the baseline (km^2^, also expressed as a percentage value: e.g. +13% increase compared to baseline). The* ***red line*** *enables the visual comparison of the blue bar with the error bars. The* ***error bars*** *show the change in the decadal minimum, mean and maximum as a result of changing just one driver, e.g. the sea level rise, and keeping all other drivers constant. (Abbreviations: ‘actual’: decadal mean change compared to baseline, ‘PTRO’: precipitation, temperature and runoff linked to different climate scenarios, ‘rSLR’: relative sea level rise, ‘Cyc’: cyclone frequency, ‘Pold’: polder maintenance, ‘Pop’: population size, ‘Econ’: economic changes at household level such as selling price of crops, cost of food, etc., ‘LC’: land cover, ‘FP’: farming practices)*

*
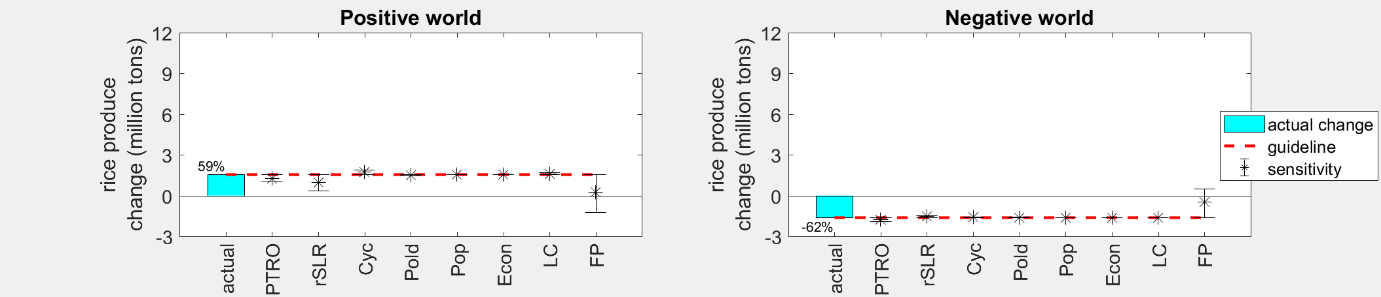
*

*Figure S13: Sensitivity of the rice productivity output to different drivers within the Positive and Negative world scenarios at mid-Century. The* ***‘actual change’*** *blue bar shows the expected mean decadal change in inundated area compared to the baseline (km^2^, also expressed as a percentage value: e.g. +13% increase compared to baseline). The* ***red line*** *enables the visual comparison of the blue bar with the error bars. The* ***error bars*** *show the change in the decadal minimum, mean and maximum as a result of changing just one driver, e.g. the sea level rise, and keeping all other drivers constant. (Abbreviations: ‘actual’: decadal mean change compared to baseline, ‘PTRO’: precipitation, temperature and runoff linked to different climate scenarios, ‘rSLR’: relative sea level rise, ‘Cyc’: cyclone frequency, ‘Pold’: polder maintenance, ‘Pop’: population size, ‘Econ’: economic changes at household level such as selling price of crops, cost of food, etc., ‘LC’: land cover, ‘FP’: farming practices)*

*
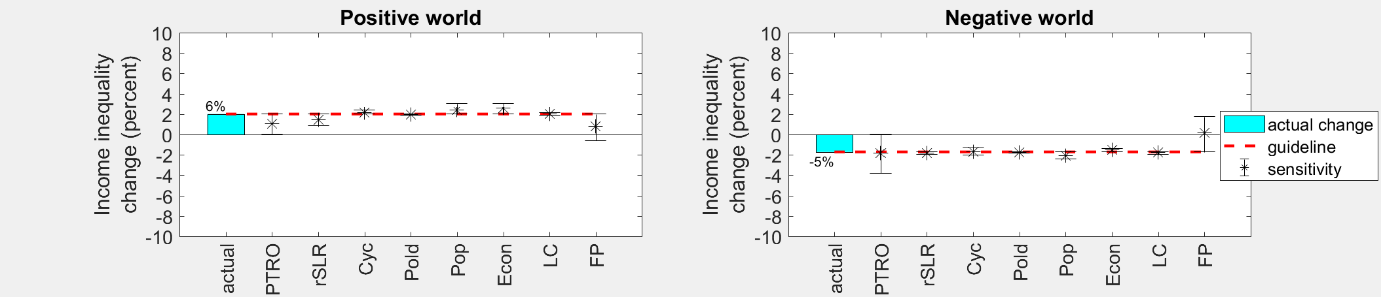
*

*Figure S14: Sensitivity of the income inequality (GINI coefficient) output to different drivers within the Positive and Negative world scenarios at mid-Century. The* ***‘actual change’*** *blue bar shows the expected mean decadal change in inundated area compared to the baseline (km^2^, also expressed as a percentage value: e.g. +13% increase compared to baseline). The* ***red line*** *enables the visual comparison of the blue bar with the error bars. The* ***error bars*** *show the change in the decadal minimum, mean and maximum as a result of changing just one driver, e.g. the sea level rise, and keeping all other drivers constant. (Abbreviations: ‘actual’: decadal mean change compared to baseline, ‘PTRO’: precipitation, temperature and runoff linked to different climate scenarios, ‘rSLR’: relative sea level rise, ‘Cyc’: cyclone frequency, ‘Pold’: polder maintenance, ‘Pop’: population size, ‘Econ’: economic changes at household level such as selling price of crops, cost of food, etc., ‘LC’: land cover, ‘FP’: farming practices)*

*
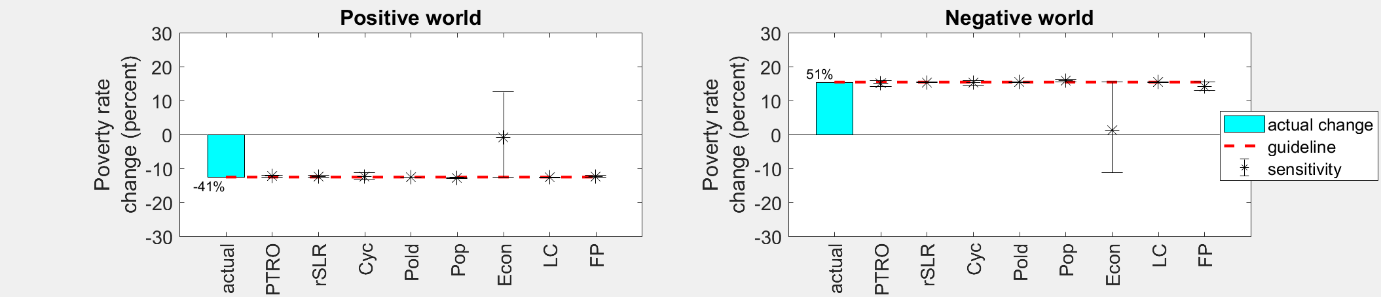
*

*Figure S15: Sensitivity of the Cost of Basic Needs (Upper Poverty Line)-based poverty rate output to different drivers within the Positive and Negative world scenarios at mid-Century. The* ***‘actual change’*** *blue bar shows the expected mean decadal change in inundated area compared to the baseline (km^2^, also expressed as a percentage value: e.g. +13% increase compared to baseline). The* ***red line*** *enables the visual comparison of the blue bar with the error bars. The* ***error bars*** *show the change in the decadal minimum, mean and maximum as a result of changing just one driver, e.g. the sea level rise, and keeping all other drivers constant. (Abbreviations: ‘actual’: decadal mean change compared to baseline, ‘PTRO’: precipitation, temperature and runoff linked to different climate scenarios, ‘rSLR’: relative sea level rise, ‘Cyc’: cyclone frequency, ‘Pold’: polder maintenance, ‘Pop’: population size, ‘Econ’: economic changes at household level such as selling price of crops, cost of food, etc., ‘LC’: land cover, ‘FP’: farming practices)*

*
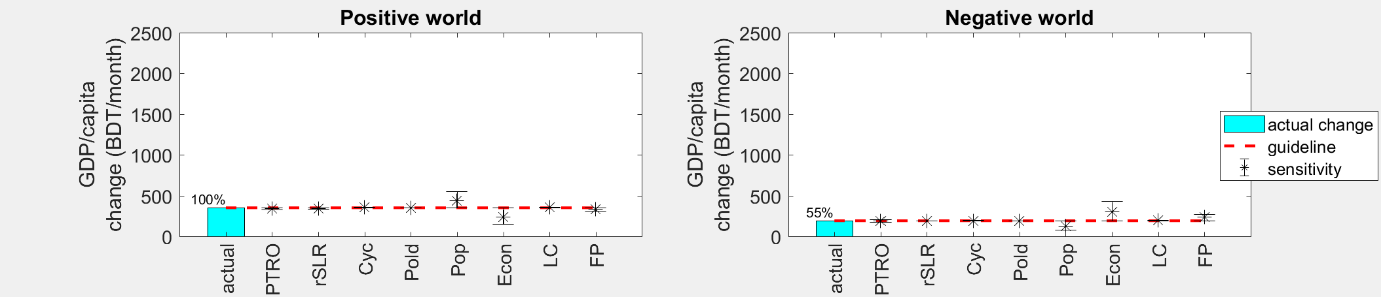
*

*Figure S16: Sensitivity of the GDP/capita output to different drivers within the Positive and Negative world scenarios at mid-Century. The* ***‘actual change’*** *blue bar shows the expected mean decadal change in inundated area compared to the baseline (km^2^, also expressed as a percentage value: e.g. +13% increase compared to baseline). The* ***red line*** *enables the visual comparison of the blue bar with the error bars. The* ***error bars*** *show the change in the decadal minimum, mean and maximum as a result of changing just one driver, e.g. the sea level rise, and keeping all other drivers constant. (Abbreviations: ‘actual’: decadal mean change compared to baseline, ‘PTRO’: precipitation, temperature and runoff linked to different climate scenarios, ‘rSLR’: relative sea level rise, ‘Cyc’: cyclone frequency, ‘Pold’: polder maintenance, ‘Pop’: population size, ‘Econ’: economic changes at household level such as selling price of crops, cost of food, etc., ‘LC’: land cover, ‘FP’: farming practices)*

***Table S1****: Details of the input scenarios*

This paper builds extensively on the scenario narratives developed in the ESPA Deltas project (Allan and Barbour 2015, Allan et al. 2018, Allan et al. 2022) and the historic trends that are observed in coastal Bangladesh (Hossain et al. 2016). Thirty-six basic simulations were run by using all combinations of the following climate, sea-level rise, polder maintenance and socio-economic development input scenarios (Figure 2). The main analysis of the paper only focuses on a Positive World and a Negative World scenario to quantify the system sensitivities in two opposing scenarios (Figures 3-4). The one-at-a-time (i.e. local) sensitivity analysis of the Positive and Negative world scenarios varied every input type one-by-one (keeping all other inputs at the same value).

The simulations started in 1985 and run until 2050. We used the 1985-2004 period as a spin-up period to allow the model to settle. The 2005-2014 period was used as the baseline and the analysis focused on the 2015-2050 period. Changes presented are therefore between 2005-2014 and 2050.

**Climate**: The UK MetOffice HadRM3/PRECIS Regional Climate Model scenarios based on the SRES A1B emissions scenario (which is similar to RCP 6.0) (Caesar et al. 2015) are used for the precipitation, air temperature and potential evapotranspiration inputs of ΔDIEM. The magnitude of climate change to 2050 is only weakly coupled to future emissions and the divergence of climate scenarios aremore apparent after 2050 (IPCC 2021). In this application, the following three climate time series are used:

1. the Q8 ensemble member of the Hadley RCM (compared to mean of 2000-2015: +1.78^o^C temperature increase, +1.51^o^C in monsoon period, +2.22^o^C in dry season period; -23% annual precipitation, -20% total monsoon rain, -53% total dry season rainfall) – used in the “Positive World” in Table 1;
2. the Q16 ensemble member of the Hadley RCM (compared to mean of 2000-2015: +1.89^o^C temperature increase, +1.45^o^C in monsoon period, +2.51^o^C in dry season period; -4% annual precipitation, -2% total monsoon rain, -16% total dry season rainfall) – used in the “Negative World” in Table 1;
3. a time series, where ‘no climate change’ occurs (the Q8 scenario’s 2001-2010 climate and run-off data is looped throughout the simulation period).

|  | **2000-15** | | | **2040-55** | | | **Change** | | |
| --- | --- | --- | --- | --- | --- | --- | --- | --- | --- |
|  | Annual | Dry season | Monsoon | Annual | Dry season | Monsoon | Annual | Dry season | Monsoon |
| **Q8** | **Temperature (^o^C)** | | | | | | **Absolute change (^o^C)** | | |
| Min | 25.08 | 20.76 | 27.62 | 26.58 | 22.57 | 28.73 | 1.50 | 1.81 | 1.11 |
| Mean | 26.15 | 22.02 | 28.39 | 27.93 | 24.23 | 29.90 | **1.78** | **2.22** | **1.51** |
| Max | 26.91 | 23.48 | 29.52 | 28.88 | 25.60 | 31.17 | 1.97 | 2.12 | 1.66 |
| St dev | 0.47 | 0.72 | 0.49 | 0.64 | 0.82 | 0.71 | 0.17 | 0.10 | 0.22 |
| **Q16** | **Temperature (^o^C)** | | | | | | **Absolute change (^o^C)** | | |
| Min | 26.96 | 22.71 | 28.87 | 28.58 | 25.01 | 30.38 | 1.62 | 2.31 | 1.51 |
| Mean | 27.43 | 23.43 | 29.76 | 29.33 | 25.95 | 31.21 | **1.89** | **2.51** | **1.45** |
| Max | 28.76 | 24.55 | 31.39 | 30.23 | 27.35 | 32.05 | 1.48 | 2.80 | 0.67 |
| St dev | 0.47 | 0.46 | 0.63 | 0.42 | 0.73 | 0.38 | -0.05 | 0.27 | -0.25 |
| **Q8** | **Precipitation (mm)** | | | | | | **Percent change** | | |
| Min | 1,453 | 34 | 1,075 | 1,290 | 30 | 1,054 | -11.20 | -10.97 | -1.90 |
| Mean | 2,099 | 215 | 1,672 | 1,623 | 100 | 1,345 | **-22.69** | **-53.18** | **-19.57** |
| Max | 2,769 | 632 | 1,972 | 2,146 | 250 | 1,949 | -22.51 | -60.40 | -1.16 |
| St dev | 363 | 153 | 268 | 271 | 62 | 251 | -25.43 | -59.56 | -6.13 |
| **Q16** | **Precipitation (mm)** | | | | | | **Percent change** | | |
| Min | 897 | 26 | 835 | 1,205 | 14 | 954 | 34.35 | -45.00 | 14.19 |
| Mean | 1,623 | 88 | 1,433 | 1,566 | 74 | 1,405 | **-3.50** | **-16.18** | **-1.94** |
| Max | 2,408 | 164 | 2,324 | 2,305 | 174 | 2,148 | -4.25 | 5.77 | -7.57 |
| St dev | 421 | 41 | 397 | 320 | 53 | 349 | -24.07 | 28.91 | -12.20 |

*Note: Dry season: Nov-Mar; Monsoon Jun-Oct*

Looking beyond 2050 to 2100, these climate trends continue, and the Positive World becomes warmer and moderately drier and the Negative World becomes warmer and wetter with more extremes.

**Sea-level rise** projections are based on the Fifth Assessment Report of the Intergovernmental Panel on Climate Change (Church et al. 2013):

1. low SLR: 26cm in 2050 + a uniform subsidence of 2.5mm/year (relative SLR: 38.5cm);
2. high SLR: 61cm in 2050 + a uniform subsidence of 2.5mm/year (relative SLR: 73.5cm).

The high SLR scenario comprises a high impact, low likelihood (i.e., high-end) SLR scenario (Nicholls et al. 2021a).

The estimates of subsidence are consistent with the observations of Brown and Nicholls (2015) and Becker et al. (2020) which are assumed to continue to 2050. We note that unlike in some other deltas there is currently no systematic evidence of enhanced delta subsidence in Bangladesh (Nicholls et al. 2021b).

**Cyclone frequencies** of the simulation were applied as plausible scenarios of climate-induced shocks. The basic cyclone sequence was derived from historic cyclone frequency and landfall location (Alam and Dominey-Howes 2015, Seneviratne et al. 2021, Khan et al. 2022). Based on the recorded cyclones for the period of 1916-2009, the SIDR-type cyclones (140+ km/h wind speed) are expected with a return frequency of 0.4 cyclone per decade, whereas AILA-type cyclones (80-140 km/h wind speed) has a return period of 4.2 per decade. The cyclone tracks were grouped into five landfall locations from west to east: West Bengal (India) (west of study area), Sundarbans, Barguna/Patuakhali, Bhola and Meghna (east of study area). Where cyclone tracks were available, it was also visually checked against the NOAAA database (<https://www.coast.noaa.gov/hurricanes/>). Simulated sequences, with modified (higher and lower) cyclone frequencies in the sensitivity analyses (Table S2), giving three cyclone sequences. Future cyclones years, cyclone strengths and landfall locations, were randomly selected based on the return periods for three scenario cases as defined in Table S2:

1. cyclones occur with the observed frequency, magnitude and landfall locations in the future;
2. cyclone frequency increases (+50%);
3. cyclone frequency decreases (-50%).

**Embankment maintenance** scenarios were uniformly applied to the embankments around all the polders of the study area:

1. maintain existing embankment heights (at design height) and current drainage infrastructure within each polder;
2. embankments are not maintained and progressively deteriorate, losing effective height (-3cm / year) and exposing the area within the polder to a greater chance of inundation.

This implies integrated policies, effective implementation and good maintenance in a Positive World with well managed and maintained coastal and drainage infrastructure, effective groundwater management and implementation of land zoning. In contrast, in a Negative World there is poor planning and implementation of delta management interventions and low capacity for maintenance which is expressed in the input as a loss of effective height.

**Socio-economic development** of coastal Bangladesh was characterised within the ESPA Deltas project with experts and a series of stakeholder workshops over 4 years (Allan et al. 2018, Allan et al. 2022). These scenarios combine demographic changes, land cover and land use changes, and economic changes. Each of these aspects are summarised below.

**Population size** based on the 2011 CENSUS is ca. 14 million (BBS et al. 2012). In this application three stylised scenarios are tested based on Szabo et al. (2016):

1. population size is constant at 14 million to 2050 (Positive World);
2. population size is constant at 14 million until 2030 and then a gradually decline to 12.85 million by 2050;
3. the population gradually declines from today to 11.2 million by 2050 (Negative World).

**Economic development trends** were analysed and plausible future trends identified by Hunt (2018). Economic growth in Bangladesh has been >5% pa sustained over two decades and 7.8% per annum in 2018 (World Bank 2020). But based on analogues from other countries, such a high growth rate is not projected to be sustained in the long-term (Riahi et al. 2017), and hence we use a lower value to 2050. Our simulation also recognises that the economies of deltas are evolving away from agriculture and fisheries to manufacturing and services based on observations (Cazcarro et al. 2018, Arto et al. 2020). Hunt also approximated the effect of these trends on the coastal households (wages, market price, food cost, livelihood costs, wages, etc.):

1. Fast growth: Manufacturing and services: 87% by 2050 (2.5%/yr); Agriculture: 45% by 2050 (1.3%/yr); Household expenses: 45% by 2050 (1.3%/yr); Wages: 70% by 2050 (2%/yr) (Positive World).
2. Moderate growth: Manufacturing and services 45% by 2050 (1.3%/yr); Agriculture: 24% by 2050, Household expenses: 24% by 2050, Wages: 24% by 2050 (0.62%/yr).
3. Slow growth: Manufacturing and services 24% by 2050 (0.62%/yr); Agriculture: 0% by 2050, Household expenses: 0% by 2050, Wages: 0% by 2050 (Negative World).

**Land cover** changes are assumed based on stakeholder scenario narratives about the future. The 2010 land cover observation (Mukhopadhyay et al. 2018) is modified with the following percentage changes:

1. Agriculture area slightly decreases (-5%) to increase mangrove area. Sundarbans loses 5% area to aquaculture and agriculture. The land zoning proposal of the Ministry of Agriculture is promoted and 30% of land cover area is converted accordingly (MA and FAO 2013) (Positive World).
2. 10% of Sundarbans area and 5% of other mangrove areas are converted to agriculture and aquaculture.
3. 20 % of Sundarbans area and 10% of other mangrove areas are converted to agriculture and aquaculture. Agriculture area is reduced by 20% if salinity is high (>12 dS/m) and by 10% if salinity is moderately high (8-12 dS/m) (Negative World).

**Farming practice** scenarios follow the observed patterns:

1. Current cropping patterns continue with improved varieties. New crop types increase production (+20%). Salinity tolerance of rice increases from 6 dS/m (2015) to 13 dS/m (2050) (Positive World).
2. Current cropping patterns continue with current crop varieties.
3. Traditional crop types and cropping patterns are assumed with neither yield (0%) nor salinity tolerance (6 dS/m) improvements (Negative World).

Note that research and development on crop varieties is ongoing in Bangladesh (BARC 2013) and this has resulted in crop varieties that are more resilient to salinity. In the Positive World, these trends are expected to continue for which there was strong stakeholder support in our consultations.

***Table S2****: List of randomly selected, simulated cyclones*

The basic cyclone sequence applied in the simulations was derived from historic cyclone frequency and landfall location(Alam and Dominey-Howes 2015, Seneviratne et al. 2021, Khan et al. 2022), with modified (higher and lower) cyclone frequencies in the sensitivity analyses. Below the cyclone strengths (following historical storms), cyclone landfall location and date of landfall in the simulations are provided, including scenarios with an increase and decrease of frequency.

**Strength: Aila; Landfall location: Barguna/Patuakhali (historic track)**

cyclones occur with the observed frequency, magnitude and landfall locations: 23/05/2009, 23/10/2026, 23/11/2028

cyclone frequency increases (+50%): 23/05/2009, 23/05/2016, 23/11/2049

cyclone frequency decreases (-50%): 23/05/2009, 23/05/2025

**Strength: Aila; Landfall location: Bhola**

cyclones occur with the observed frequency, magnitude and landfall locations: 23/09/2035, 23/10/2048

cyclone frequency increases (+50%): 23/10/2012, 23/10/2017, 23/11/2020, 23/11/2043

cyclone frequency decreases (-50%): 23/05/2045

**Strength: Aila; Landfall location: Meghna**

cyclones occur with the observed frequency, magnitude and landfall locations:23/05/2031, 23/05/2046

cyclone frequency increases (+50%): 23/11/2021, 23/04/2025

cyclone frequency decreases (-50%): 23/05/2048

**Strength: Aila; Landfall location: Sundarbans**

cyclones occur with the observed frequency, magnitude and landfall locations: 23/11/2013, 23/05/2016, 23/05/2019, 23/05/2041, 23/11/2044, 23/10/2050

cyclone frequency increases (+50%): 23/11/2011, 23/10/2018, 23/05/2031, 23/05/2036, 23/09/2038, 23/05/2041, 23/05/2042, 23/08/2043, 23/05/2045

cyclone frequency decreases (-50%): 23/09/2012, 23/05/2027, 23/08/2031

**Strength: Aila; Landfall location: West Bengal**

cyclones occur with the observed frequency, magnitude and landfall locations: 23/05/2024, 23/05/2025, 23/11/2027, 23/10/2047

cyclone frequency increases (+50%): 11/05/2020, 11/05/2024, 11/05/2030, 11/11/2034, 11/05/2040, 11/11/2042

cyclone frequency decreases (-50%): 23/11/2015, 11/11/2036

**Strength: Sidr; Landfall location: Barguna/Patuakhali (historic track)**

cyclones occur with the observed frequency, magnitude and landfall locations: 11/11/2007

cyclone frequency increases (+50%): 11/11/2007, 23/05/2014, 23/10/2016

cyclone frequency decreases (-50%): 11/11/2007, 23/11/2037

**Strength: Sidr; Landfall location: Bhola**

cyclones occur with the observed frequency, magnitude and landfall locations: *none*

cyclone frequency increases (+50%): 23/05/2038

cyclone frequency decreases (-50%): *none*

**Strength: Sidr; Landfall location: Sundarbans**

cyclones occur with the observed frequency, magnitude and landfall locations: 11/10/2029

cyclone frequency increases (+50%): 23/05/2021, 23/06/2022

cyclone frequency decreases (-50%): 23/09/2049

***References***

Adams, H., W. N. Adger, S. Ahmad, A. Ahmed, A. Begum, et al. (2016a). "Spatial and temporal dynamics of multidimensional well-being, livelihoods and ecosystem services in coastal Bangladesh." Scientific Data **3**: 160094.

Adams, H., W. N. Adger, S. Ahmad, A. Ahmed, D. Begum, et al. (2016b). Spatial and temporal dynamics of multidimensional well-being, livelihoods and ecosystem services in coastal Bangladesh. Colchester, Essex: UK Data Archive. 10.5255/UKDA-SN-852179.

Alam, E. and D. Dominey-Howes (2015). "A new catalogue of tropical cyclones of the northern Bay of Bengal and the distribution and effects of selected landfalling events in Bangladesh." International Journal of Climatology **35**(6): 801-835.

Allan, A. and E. Barbour (2015). Building Scenarios for Ecosystem Services and Poverty Alleviation - the ESPA Deltas Approach, ESPA Deltas Working Paper, available at <http://www.espadelta.net>.

Allan, A., M. Lim and E. J. Barbour (2018). Chapter 10: Incorporating stakeholder perspectives in scenario developmen. Ecosystem Services For Well-Being In Deltas: Integrated Assessment For Policy Analysis. R. J. Nicholls, C. W. Hutton, W. N. Adger et al., Palgrave, ISBN 978-3-319-71092-1.

Allan, A. A., E. Barbour, R. J. Nicholls, C. Hutton, M. Lim, et al. (2022). "Developing socio-ecological scenarios: A participatory process for engaging stakeholders." Science of The Total Environment **807**: 150512.

Allen, R. G., L. S. Pereira, D. Raes and M. Smith (1998). FAO Irrigation and Drainage Paper - No. 56: Crop Evapotranspiration (guidelines for computing crop water requirements), FAO, Water Resources, Development and Management Service, Rome, Italy.

Amoako Johnson, F., C. W. Hutton, D. Hornby, A. N. Lázár and A. Mukhopadhyay (2016). "Is shrimp farming a successful adaptation to salinity intrusion? A geospatial associative analysis of poverty in the populous Ganges–Brahmaputra–Meghna Delta of Bangladesh." Sustainability Science **11**(3): 423-439.

Arto, I., I. Cazcarro, A. Markandya, S. Hazra, R. N. Bhattacharya, et al. (2020). Delta Economics and Sustainability. Deltas in the Anthropocene. R. J. Nicholls, W. N. Adger, C. W. Hutton and S. E. Hanson. Cham, Springer International Publishing**:** 179-200.

BARC (2013). Appropriate Agricultural Technology for Southern Region (report)**:** 228 pages.

BBS (1991). Report of the Household Income & Expenditure Survey 1991, Bangladesh Bureau of Statistics, Statistical Division, Ministry of Planning.

BBS, SID and MP (2012). Population and Housing Census 2011. Socio-economic and demographic report. National Series - volume 4, Bangladesh Bureau of Statistics, Statistics and Informatics Division, Ministry of Planning

Becker, M., F. Papa, M. Karpytchev, C. Delebecque, Y. Krien, et al. (2020). "Water level changes, subsidence, and sea level rise in the Ganges–Brahmaputra–Meghna delta." Proceedings of the National Academy of Sciences **117**(4): 1867-1876.

Brown, S. and R. J. Nicholls (2015). "Subsidence and human influences in mega deltas: The case of the Ganges–Brahmaputra–Meghna." Science of The Total Environment **527-528**: 362-374.

Caesar, J., T. Janes, A. Lindsay and B. Bhaskaran (2015). "Temperature and precipitation projections over Bangladesh and the upstream Ganges, Brahmaputra and Meghna systems." Environmental Science: Processes & Impacts **17**(6): 1047-1056.

Cazcarro, I., I. Arto, S. Hazra, R. N. Bhattacharya, P. Osei-Wusu Adjei, et al. (2018). "Biophysical and Socioeconomic State and Links of Deltaic Areas Vulnerable to Climate Change: Volta (Ghana), Mahanadi (India) and Ganges-Brahmaputra-Meghna (India and Bangladesh)." Sustainability **10**: 893.

Church, J. A., P. U. Clark, A. Cazenave, J. M. Gregory, S. Jevrejeva, et al. (2013). Sea level change. Climate change 2013: The physical science basis. Contribution of Working Group I to the Fifth Assessment Report of the Intergovernmental Panel on Climate Change. T. F. Stocker, D. Qin, G.-K. Plattner et al. Cambridge, United Kingdom and New York, NY, USA: Cambridge University Press.

Clarke, D., S. Williams, M. Jahiruddin, K. Parks and M. Salehin (2015). "Projections of on-farm salinity in coastal Bangladesh." Environmental Science: Processes & Impacts **17**(6): 1127-1136.

Dasgupta, S., M. M. Hossain, M. Huq and D. Wheeler (2015). "Climate change and soil salinity: The case of coastal Bangladesh." Ambio **44**(8): 815-826.

Falkingham, J. and C. Namazie (2002). Measuring health and poverty: a review of approaches to identifying the poor, DFID Health Systems Resource Centre**:** 70.

FarakkaTreaty (1996). Treaty between the government of the People’s Republic of Bangladesh and the government of the Republic of India on sharing of the Ganga / Ganges water at Farakka. Government of the People's Republic of Bangladesh, Dhaka.

Fernandes, J. A., S. Kay, M. A. R. Hossain, M. Ahmed, W. W. L. Cheung, et al. (2016). "Projecting marine fish production and catch potential in Bangladesh in the 21st century under long-term environmental change and management scenarios." ICES Journal of Marine Science: Journal du Conseil **73**(5): 1357-1369.

Forrester, J. W. and P. M. Senge (1980). "Tests for building confidence in system dynamic models." TIMS Studies in the Management Sciences **14**: 209-228.

Hossain, M. S., J. A. Dearing, M. M. Rahman and M. Salehin (2016). "Recent changes in ecosystem services and human well-being in the Bangladesh coastal zone." Regional Environmental Change **16**(2): 429-443.

Hunt, A. (2018). Future scenarios of economic development. Ecosystem Services For Well-Being In Deltas: Integrated Assessment For Policy Analysis. R. J. Nicholls, C. W. Hutton, W. N. Adger et al., Palgrave, ISBN 978-3-319-71092-1.

Hutton, C., R. Nicholls, A. N. Lázár, A. Chapman, M. Schaafsma, et al. (2018). "Potential Trade-Offs between the Sustainable Development Goals in Coastal Bangladesh." Sustainability **10**(4): 1108.

IPCC (2021). Summary for Policymakers. Climate Change 2021: The Physical Science Basis. Contribution of Working Group I to the Sixth Assessment Report of the Intergovernmental Panel on Climate Change. V. Masson-Delmotte, P. Zhai, A. Pirani et al. Cambridge University Press, Cambridge, United Kingdom and New York, NY, USA**:** 3−32.

Khan, M. J. U., F. Durand, K. Emanuel, Y. Krien, L. Testut, et al. (2022). "Storm surge hazard over Bengal delta: a probabilistic–deterministic modelling approach." Nat. Hazards Earth Syst. Sci. **22**(7): 2359-2379.

Lázár, A. N., H. Adams, W. N. Adger and R. J. Nicholls (2020). "Modelling household well-being and poverty trajectories: An application to coastal Bangladesh." PLOS ONE **15**(9): e0238621.

Lázár, A. N., D. Clarke, H. Adams, A. R. Akanda, S. Szabo, et al. (2015). "Agricultural livelihoods in coastal Bangladesh under climate and environmental change - a model framework." Environmental Science: Processes & Impacts **17**(6): 1018-1031.

Lázár, A. N., A. Payo, H. Adams, A. Ahmed, A. Allan, et al. (2018). Integrative analysis applying the Delta Dynamic Integrated Emulator Model in south-west coastal Bangladesh. Ecosystem Services For Well-Being In Deltas: Integrated Assessment For Policy Analysis. R. J. Nicholls, C. W. Hutton, W. N. Adger et al., Palgrave, ISBN 978-3-319-71092-1.

Lázár, A. N., A. Payo and R. J. Nicholls (2019). Delta Dynamic Integrated Emulator Model version 1.6 (19 April 2018). University of Southampton, UK.

MA and FAO (2013). Master Plan for Agriculture Development In the Southern Region of Bangladesh, Ministry of Agriculture of the Government of the People's Republic of Bangladesh, Food and Agriculture Organisation of the United Antions.

MathWorks (2017). MATLAB and Statistics Toolbox Release 2017a, The MathWorks, Inc., Natick, Massachusetts, United States.

Mukhopadhyay, A., D. Hornby, C. W. Hutton, A. N. Lázár, F. A. Johnson, et al. (2018). Chapter 20: Land cover and land use analysis in coastal Bangladesh. Ecosystem Services For Well-Being In Deltas: Integrated Assessment For Policy Analysis. R. J. Nicholls, C. W. Hutton, W. N. Adger et al., Palgrave, ISBN 978-3-319-71092-1.

Mukhopadhyay, A., P. Mondal, J. Barik, S. M. Chowdhury, T. Ghosh, et al. (2015). "Changes in mangrove species assemblages and future prediction of the Bangladesh Sundarbans using Markov chain model and cellular automata." Environmental Science: Processes & Impacts **17**(6): 1111-1117.

Nicholls, R. J., S. E. Hanson, J. A. Lowe, A. B. A. Slangen, T. Wahl, et al. (2021a). "Integrating new sea-level scenarios into coastal risk and adaptation assessments: An ongoing process." WIREs Climate Change **12**(3): e706.

Nicholls, R. J., C. W. Hutton, W. N. Adger, S. Hanson, M. Rahaman, et al. (2018). Ecosystem Services For Well-Being In Deltas: Integrated Assessment For Policy Analysis, Palgrave , ISBN 978-3-319-71092-1.

Nicholls, R. J., C. W. Hutton, A. N. Lázár, A. Allan, W. N. Adger, et al. (2016). "Integrated assessment of social and environmental sustainability dynamics in the Ganges-Brahmaputra-Meghna delta, Bangladesh." Estuarine and coastal shelf science **183**: 370–381.

Nicholls, R. J., D. Lincke, J. Hinkel, S. Brown, A. T. Vafeidis, et al. (2021b). "A global analysis of subsidence, relative sea-level change and coastal flood exposure." Nature Climate Change **11**(4): 338-342.

Payo, A., A. N. Lázár, D. Clarke, R. J. Nicholls, L. Bricheno, et al. (2017). "Modeling daily soil salinity dynamics in response to agricultural and environmental changes in coastal Bangladesh." Earth's Future **5**: 495–514.

Payo, A., A. Mukhopadhyay, S. Hazra, T. Ghosh, S. Ghosh, et al. (2016). "Projected changes in area of the Sundarban mangrove forest in Bangladesh due to SLR by 2100." Climatic Change **139**(2): 279-291.

Rahman, M. M., R. J. Nicholls, S. Hanson, M. Salehin and S. Alam (2019). Integrated assessment of the Bangladesh Delta Plan 2100. Analysis of selected interventions., BUET; Southampton University; GED, Planning Commission, People’s Republic of Bangladesh; ISBN: 978-984-34-7513-8; <https://iwfm.buet.ac.bd/site/wp-content/uploads/2020/01/Book-2019-Integrated-Assessment-BDP-2100-ESPA-Deltas-1.pdf>.

Riahi, K., D. P. van Vuuren, E. Kriegler, J. Edmonds, B. C. O’Neill, et al. (2017). "The Shared Socioeconomic Pathways and their energy, land use, and greenhouse gas emissions implications: An overview." Global Environmental Change **42**: 153-168.

Seneviratne, S. I., X. Zhang, M. Adnan, W. Badi, C. Dereczynski, et al. (2021). Weather and Climate Extreme Events in a Changing Climate. Climate Change 2021: The Physical Science Basis. Contribution of Working Group I to the Sixth Assessment Report of the Intergovernmental Panel on Climate Change. V. Masson-Delmotte, P. Zhai, A. Pirani et al. Cambridge University Press, Cambridge, United Kingdom and New York, NY, USA**:** 1513–1766.

Szabo, S., D. Begum, S. Ahmad, Z. Matthews and P. K. Streatfield (2016). "Scenarios of population change in the coastal Ganges Brahmaputra Delta (2011-2051)." Asia-Pacific Population Journal **30**(2): 51-72.

Whitehead, P. G., E. Barbour, M. N. Futter, S. Sarkar, H. Rodda, et al. (2015). "Impacts of climate change and socio-economic scenarios on flow and water quality of the Ganges, Brahmaputra and Meghna (GBM) river systems: low flow and flood statistics." Environmental Science: Processes & Impacts **17**(6): 1057-1069.

World Bank. (2020). "Data portal: GDP growth (annual %) - Bangladesh." Retrieved 14/01/2020, 2020, from <https://data.worldbank.org/indicator/NY.GDP.MKTP.KD.ZG?locations=BD>.
